# Induction of neutralising antibodies against conserved rhinovirus capsid protein VP4 is dependent on presentation of VP4 peptide immunogens in a virus-like conformation

**DOI:** 10.1371/journal.ppat.1014416

**Published:** 2026-07-16

**Authors:** James T. Kelly, Giann Kerwin Y. Dellosa, Joseph Newman, Adel A. M. Mohamed, Julia Aniscenko, Sebastian L. Johnston, Jason Schnell, Tobias J. Tuthill

**Affiliations:** 1 The Pirbright Institute, Pirbright, United Kingdom; 2 National Heart and Lung Institute, Imperial College London, London, United Kingdom; 3 Department of Biochemistry, University of Oxford, Oxford, United Kingdom; Duke-National University of Singapore, SINGAPORE

## Abstract

Infection with rhinovirus (RV) is a frequent cause of the common cold and is also associated with more significant morbidity, including hospitalisation, in people with chronic lung disease. At present, there is no licensed RV vaccine or antiviral. There are over 150 RV strains classified into 3 species (RV-A, RV-B, RV-C), and approximately 100 of these have been characterised as distinct serotypes. There is no cross-protection between serotypes because of high diversity in immunodominant antigenic sites. This makes it impractical to create a broadly protective vaccine using traditional methods, involving whole capsids. An alternative strategy is to direct the immune response towards conserved but less dominant epitopes. The capsid protein VP4 is highly conserved within each RV species, and antibodies that target the N-terminal region of VP4 have previously been shown to be neutralising. Here, we investigate the immunogenicity of three RV N-terminal VP4 peptides of different sizes (spanning residues 1–15, 1–30, and 1–45) presented on SpyCatcher virus-like particles (VLPs). Neutralising antibodies were only induced against the shortest peptide. The neutralising epitope contained within the first 15 amino acids was antigenically and structurally different in the context of longer peptides. This indicates the conformation of the 1–15 region of VP4 is critical for inducing antibodies that neutralise by binding VP4 in the context of the virus particle. Therefore, correctly displayed VP4 peptides can recapitulate virus-like antigenicity. These findings improve our understanding of VP4 antigenicity and may inform the design of future RV vaccines. This work could also impact the design of other peptide vaccines, since a variable antigenic conformation is a common characteristic of pathogen-derived peptide targets.

## Author summary

Rhinoviruses (RVs) are highly prevalent respiratory viruses that frequently cause common cold symptoms. For people with chronic lung disease such as asthma, infection can cause significant worsening of disease, leading to increased suffering and hospitalisation. There are currently no licensed vaccines or anti-viral drugs that target RV infections. Development of an RV vaccine would be of significant benefit to sufferers of chronic lung disease. Attempts to develop a broadly protective RV vaccine have been hindered by the presence of over 100 serotypes, which have little or no natural cross-reactivity. Previous studies have identified the highly conserved capsid protein VP4 as a potential antigen for inducing broadly protective antibodies. However, there has been little progress in evaluating its effectiveness as a vaccine candidate. In this study, we assessed the ability of different RV VP4 immunogens to induce neutralising antibodies in mice. We demonstrated that antigenic conformation of VP4 varies greatly between immunogens and presentation of the first 15 N-terminal residues in a virus-like conformation is important for generating neutralising antibodies. This knowledge could help to maximise the production of neutralising VP4-specific antibodies in future studies which could ultimately lead to a universal RV vaccine.

## Introduction

Rhinoviruses (RV) are frequent causative agents of the common cold. In the pre-COVID-19 era, RV was the most frequently detected virus in individuals with cold symptoms, with positivity rates of over 50% [1]. The incidence of many common respiratory virus infections was suppressed during the pandemic, but is now thought to have returned to pre-pandemic levels [2,3]. RVs predominantly cause a mild self-limiting illness in healthy individuals. However, infection is associated with significant morbidity [4], with studies showing RV was present in 42% of hospitalisations due to respiratory tract infections [5]. This is especially problematic for those with chronic lung diseases such as asthma [6,7] and chronic obstructive pulmonary disease (COPD) [8,9]. Additionally, RV infections have significant economic consequences, particularly for children’s education. A recent study revealed that nearly a quarter (24%) of individuals with RV-associated acute upper respiratory infections missed at least one day of work or school [10]. Furthermore, studies from the early 2000’s estimate the common cold costs the US economy between $25-$40 billion annually through lost productivity and direct health care costs [11,12]. Therefore, reducing the morbidity and spread of RV would be beneficial to sufferers of lung disease and the economy. At present, there is no approved RV vaccine or antiviral [13].

RVs are members of the enterovirus genus in the picornavirus family. There are 3 RV species known as RV-A, RV-B and RV-C. RV species are further distinguished by receptor usage. RV-A uses either intercellular adhesion molecule-1 (ICAM-1) or low-density lipoprotein receptor (LDLR). RV-B uses ICAM-1. RV-C uses cadherin-related family member 3 (CDHR3) [14]. Amongst these species there are over 100 serotypes, which have little or no natural cross-reactivity [13]. Attempts to generate cross-reactive protective/neutralising responses using inactivated virus vaccines have only been successful by immunisation with a multivalent vaccine, with one such study successfully creating a 50-valent response in rhesus macaques [15]. However, these vaccines do not target a conserved epitope, so the observed neutralisation in multivalent RV vaccines is predominantly limited to the serotypes found in the vaccine formulation [15–17]. This means that an effective multivalent RV vaccine may need to include all serotypes, which would not be feasible.

**Fig 1:**
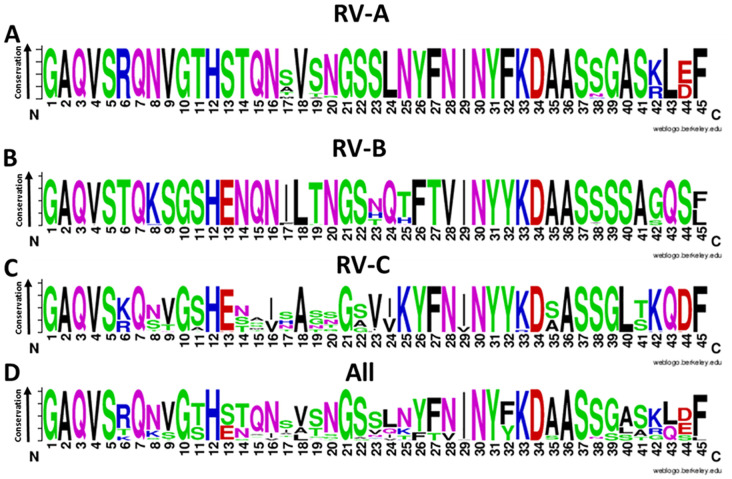
Regions of RV VP4 are highly conserved within and between RV species. Conservation of VP4 residues 1-45 between RV species A **(A)**, RV species B **(B)**, RV species C (C) and species A,B,C (D) was analysed using the WebLogo online server. Alignments were based on sequences from 77 RV-A serotypes, from 26 RV-B serotypes and 26 RV-C genotypes. All sequences were retrieved from NCBI.

There is little or no cross-protection between virus serotypes because the major neutralising immunogenic sites are formed by discontinuous epitopes on the capsid surface that are highly variable between serotypes [18]. An alternative strategy to create a broadly protective RV vaccine would therefore use a vaccine formulation that directs the immune response towards conserved epitopes. Highly conserved amino acid sequences at the N-termini of the capsid proteins VP4 and VP1 have previously been identified as linear epitopes and binding sites for neutralising antibodies. The N-terminus of VP4 is highly conserved, especially within species (Fig 1). Immunisation against full-length VP4 or peptides from VP4 has generated neutralising antibodies against several different picornaviruses, including RV, poliovirus and enterovirus A71 (EV-A71) [19–23]. RV VP4 antibodies were also shown to have broadly neutralising activity against diverse serotypes: antibodies raised against VP4 of RV-B14 neutralised RV-B14, RV-A16 and RV-A29 [19].

Structural studies demonstrate VP4 is an internal protein, buried within the capsid [24]. However, studies show that under physiological conditions, VP4 is sensitive to proteolytic digestion, where most internal components of the capsid are resistant [25]. Further studies demonstrated that VP4 antibodies can be neutralising [19]. Together, these findings indicate that although VP4 is an internal protein, it must become exposed at the capsid surface. The mechanism underlying this is that VP4 is transiently exposed on the viral surface, a process known as capsid breathing [19,25]. The mechanism of neutralisation by these VP4 antibodies is likely through the prevention of interaction between VP4 and the endosomal membrane [20]. Picornavirus VP4 plays an essential role during viral entry: when in contact with a membrane, such as an endosome, VP4 permeabilises it to form a size-selective pore [26–30]. Formation of this pore is expected to facilitate the viral genome’s exit from the capsid across the endosomal membrane and into the cytoplasm, where the genome can then replicate [26]. The N-terminus of VP4 is myristoylated; this is the covalent attachment of myristic acid, a 14-carbon saturated fatty acid [31]. This plays an important role in virus assembly and in VP4-membrane interactions [32]. Antibodies raised against the first 16 N-terminal residues of VP4 from RV-A16 can block viral pore formation in model membranes and neutralise infection [20]. Further evidence of the ability of VP4 to induce protective responses can be found in *in vivo* studies. Inoculation of suckling mice with sera from mice immunised with a hepatitis B virus core (HepB core) fusion protein containing the N-terminal 20 residues of VP4 from EV-A71 protected from EV-A71 infection [23]. Antibodies raised against peptides corresponding to the N-terminal 24 and 30 amino acids of RV-B14 VP4 and the N-terminal 20 amino acids of EV-A71 VP4 are also neutralising [19,23]. In contrast, when antibodies were raised against peptides corresponding to the C-terminal 16 amino acids of RV-A16 VP4, neutralisation was not observed [19,20,23]. These results suggest that only activity against the N-terminus of VP4 is required for neutralisation. The high level of sequence conservation at the N-terminus of VP4 (Fig 1) suggests that antibodies recognising epitopes in this region may be broadly neutralising within each RV species and potentially between species. It is not clear if myristoylation influences the antigenicity of VP4. There is also evidence that the length of the VP4 peptide influences the antigenic conformation, influencing its ability to induce neutralising antibodies [19]. This suggests the length of VP4 used in immunisations may be important to ensure that the most neutralising site is presented.

In addition to the length of the peptide, the method of presentation must also be investigated. Free peptides exhibit low immunogenicity [33], whereas displaying antigens on a VLP or carrier protein substantially enhances immunogenicity [34,35]. There are a variety of different presentation systems available, including the SpyTag/SpyCatcher system [36], which was shown to generate good immune responses to several different immunogens, including the SARS-CoV-2 receptor binding domain [37]. In this system, the VLP subunit is genetically fused to the SpyCatcher003 domain, which allows the VLP to be decorated with SpyTag-fused antigens through spontaneous formation of covalent isopeptide bonds [37]. The well-established Keyhole Limpet Hemocyanin (KLH) and HepB core assemblies for antigen presentation have already been demonstrated to induce immune responses against picornavirus VP4 peptides [19,20,23,38].

In the present study, we used the SpyTag/SpyCatcher presentation system to compare the ability of RV-A16 N-terminal VP4 peptides corresponding to amino acids 1–15, 1–30 and 1–45 to induce an immune response in mice. We included, for comparison, the use of the existing KLH presentation system. We mapped which epitopes are bound by the resulting sera and assessed their ability to neutralise RV. Here, we show that both display systems can generate an immune response to various VP4 peptides. We also determine how peptide length and display system influence the induction of neutralising antibodies.

## Results

### Different lengths of VP4 peptides can be effectively displayed on mi3 VLP via SpyTag/SpyCatcher

To create a VP4-SpyVLP vaccine candidate, VP4 peptides of different lengths were solid-phase synthesised with a run of 6 lysines as the spacer and then a C-terminal SpyTag (AHIVMVDAYKPTK) (Table 1). The lysines improve the solubility, since VP4 is very hydrophobic. VP4-SpyTag peptides were conjugated to the SpyCatcher003-mi3 VLP (SpyVLP) to generate the VP4-SpyTag:SpyCatcher003-mi3 (VP4-SpyVLP) immunogen [37,39,40]. Mi3 is a dodecahedral 60-mer nanocage, engineered from the *Thermotoga maritima* aldolase [39,41]. Six different peptides were tested, including myristoylated and non-myristoylated VP4 peptides, spanning amino acids 1–15 (1-15-VP4), 1–30 (1-30-VP4) and 1–45 (1-45-VP4) (Table 1). In the virus, the N-terminus of VP4 is covalently linked to myristic acid, a 14-carbon saturated fatty acid. However, it is unclear whether myristoylation of VP4 influences antigenicity.

**Table 1:** Peptides used in the study.

VP4 Start	VP4 End	Peptide Name	Sequence
1	15	1-15-VP4-SpyTag	**GAQVSRQNVGTHSTQ**KKKKKKGSAHIVMVDAYKPTK
1	30	1-30-VP4-SpyTag	**GAQVSRQNVGTHSTQNMVSNGSSINYFNIN**KKKKKKGSAHIVMVDAYKPTK
1	45	1-45-VP4-SpyTag	**GAQVSRQNVGTHSTQNMVSNGSSINYFNINYFKDAASSGASRLDF**KKKKKKGSAHIVMVDAYKPTK
1	15	My-1-15-VP4-SpyTag	**My-GAQVSRQNVGTHSTQ**KKKKKKGSAHIVMVDAYKPTK
1	30	My-1-30-VP4-SpyTag	**My-GAQVSRQNVGTHSTQNMVSNGSSINYFNIN**KKKKKKGSAHIVMVDAYKPTK
1	45	My-1-45-VP4-SpyTag	**My-GAQVSRQNVGTHSTQNMVSNGSSINYFNINYFKDAASSGASRLDF**KKKKKKGSAHIVMVDAYKPTK
1	15	1-15-VP4-DogTag	**GAQVSRQNVGTHSTQ**KKKKKKDIPATYEFTDGKHYITNEPIPPK
1	15	1-15-VP4-C (KLH)	**My-GAQVSRQNVGTHSTQ**KKKKKKC
1	30	1-30-VP4-C (KLH)	**My-GAQVSRQNVGTHSTQNMVSNGSSINYFNIN**KKKKKKC
1	45	1-45-VP4-C (KLH)	**My-GAQVSRQNVGTHSTQNMVSNGSSINYFNINYFKDAASSGASRLDF**KKKKKKC
5	20	5-20	**SRQNVGTHSTQNMVSN**KKKKKK
10	25	10-25	**GTHSTQNMVSNGSSIN**KKKKKK
15	30	15-30	**QNMVSNGSSINYFNIN**KKKKKK
20	35	20-35	**NGSSINYFNINYFKDA**KKKKKK
25	40	25-40	**NYFNINYFKDAASSGA**KKKKKK
30	45	30-45	**NYFKDAASSGASRLDF**KKKKKK
35	50	35-50	**AASSGASRLDFSQDPS**KKKKKK
40	55	40-55	**ASRLDFSQDPSKFTDP**KKKKKK

VP4 peptide sequences are highlighted in bold. Each peptide encoded 6x lysines (KKKKKK). SpyTag peptides included a SpyTag sequence after the lysines (GSAHIVMVDAYKPTK), and KLH peptides included a cysteine after the lysines.

**Fig 2:**
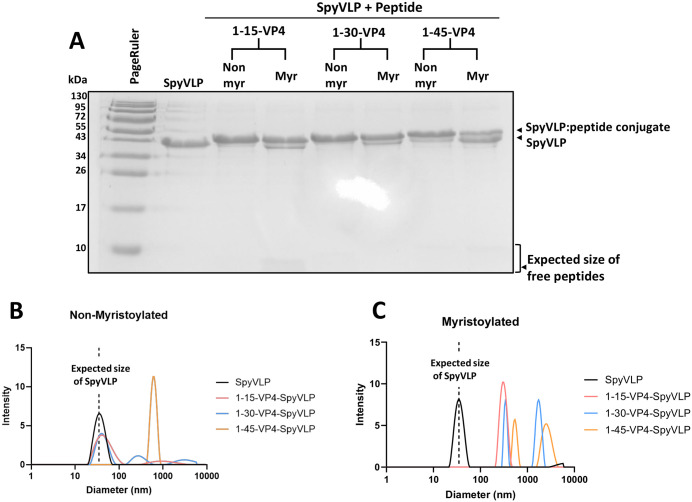
Different lengths of VP4 peptides can be effectively displayed on mi3 VLP via SpyTag/SpyCatcher. **(A)** Conjugation of SpyVLP with different SpyTag-VP4 peptides, including non-myristoylated (Non myr) and myristoylated (Myr) VP4 peptides, spanning amino acids 1-15, 1-30 and 1-45 from the N-terminus. Reactions were performed at 22 °C overnight and analysed using SDS–PAGE with Coomassie staining. Similar reactions have been repeated multiple times during subsequent preparation of material for immunisations. **(B-C)** Dynamic light scattering (DLS) characterisation of SpyVLP alone and conjugates 1-15-VP4-SpyVLP, 1-30-VP4-SpyVLP and 1-45-VP4-SpyVLP with or without myristoylation. The expected hydrodynamic diameter for SpyVLP is 37 nm.

Analysis by SDS-PAGE and Coomassie protein staining revealed that non-myristoylated peptides 1-15-VP4 and 1-30-VP4 were conjugated to SpyVLP with high efficiency, as demonstrated by the presence of a single band that is larger than the SpyVLP only control (Fig 2A). For the 1-45-VP4 non-myristoylated peptide, after conjugation, there were two prominent bands, indicating lower efficiency of conjugation (Fig 2A). All myristoylated peptides had a low conjugation efficiency as demonstrated by the presence of two prominent bands (Fig 2A).

Analysis by dynamic light scattering (DLS) showed that the SpyVLP alone had a single peak at the expected hydrodynamic diameter of 37 nm (Fig 2B). After conjugation, 1-15-VP4-SpyVLP and 1-30-VP4-SpyVLP had a major peak, which, as expected, was slightly larger with a hydrodynamic diameter of 39 nm (Fig 2B). In addition, the presence of detectable particles larger than 100 nm indicated that conjugation of these peptides to the SpyVLP had led to some aggregation (Fig 2B). In contrast, after conjugation of SpyVLP to non-myristoylated 1-45-VP4 (Fig 2B) or the myristoylated peptides 1-15-VP4, 1-30-VP4 or 1-45-VP4 (Fig 2C), the only signals detected were at a size over 100 nm. This indicated that conjugation between these peptides and the SpyVLP induced aggregation (Fig 2B, 2C). This aggregation could account for the lower conjugation efficiency observed in the myristoylated peptides and the non-myristoylated 1-45-VP4 peptide. The aggregation of the myristoylated VLP conjugates was also observed as precipitation that was visible by eye. Considering the higher conjugation efficiency, higher solubility, and lower aggregation, only conjugates of the non-myristoylated peptides were taken forward as test SpyVLP immunogens.

### Sera from mice immunised with 1-15-VP4 peptides conjugated to SpyVLP neutralise RV-A16

In addition to the novel VP4-SpyVLP peptide conjugates described above, myristoylated VP4 peptides were also displayed using the well-established KLH peptide presentation system, as used for previous RV VP4 immunisations in mice and sheep [19,20]. KLH conjugation works by conjugation to a reactive Cys group on the peptide (Table 1). Inclusion of KLH-conjugated peptides provided a control as a system previously used to generate VP4-specific responses [19,20]. KLH is prone to aggregation but such aggregation is not thought to prevent a functional response [42,43], therefore, KLH also provides a mechanism to present the myristoylated peptides in a system where potential aggregation was not problematic. Therefore, the biophysical analysis by DLS was not performed on the KLH conjugates.

**Fig 3:**
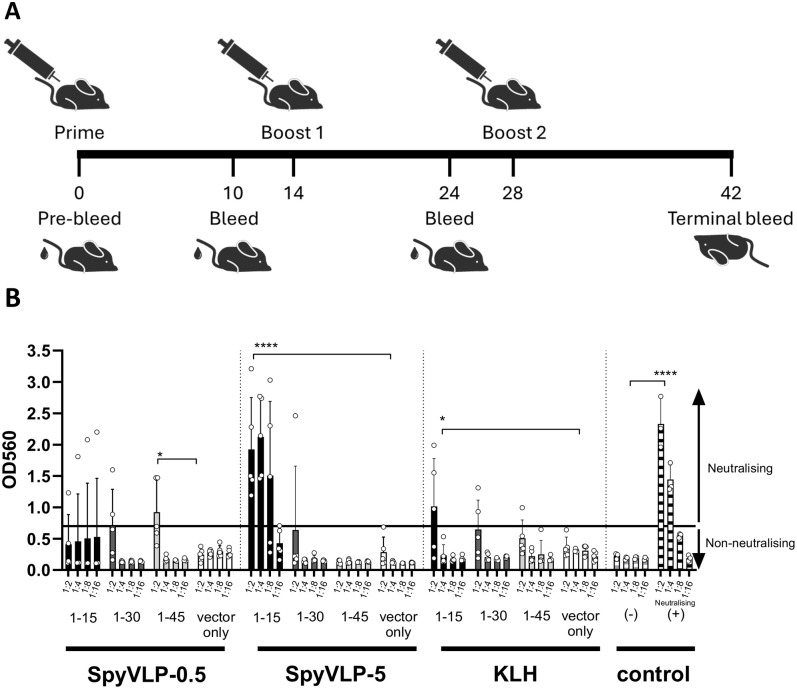
Sera from mice immunised with 1-15-VP4 peptides conjugated to SpyVLP neutralise RV-A16. **(A)** Schematic diagram of mouse immunisation schedule. **(B)** VNT was used to assess the ability of terminal bleed immune sera to neutralise RV-A16 infections in HeLa H1 cells. Serum dilutions spanned from 1:2 to 1:32. Sera were from mice immunised with peptides of different lengths (1-15, 1-30, 1-45, or vector alone) presented on SpyVLP at two different doses (SpyVLP-0.5 and SpyVLP-5) or presented on KLH. For these samples, each dot represents a different mouse (n = 5). Control samples included a PBS negative control (-) and a previously characterised anti-VP4 neutralising sheep sera (+), each dot for these control sera represents a technical replicate (n = 3). CPE was quantified by crystal violet staining and read at OD_560_. Each point corresponds to a single vaccinated mouse from n = 5 per vaccination group. Data are shown as mean ± 1 SD. Neutralisation is represented by a solid black line at OD 0.7. Statistical analysis was performed using two-way ANOVA, and post-hoc analysis was performed using Sidak’s pairwise comparison with a significance threshold of p =  0.05. Comparisons were between corresponding dilutions of sera vs mock-vaccinated controls. *p < 0.05, ***p < 0.005, ****p < 0.0005.

Mice were immunised with VP4-SpyVLP or VP4-KLH peptide conjugates, presenting VP4 amino acids 1–15, 1–30 and 1–45 from the N-terminus, as follows. Mice (n = 5) were immunised subcutaneously (SC) with either 1-15-VP4-SpyVLP (0.5 or 5 µg), 1-30 VP4-SpyVLP (0.5 or 5 µg), 1-45 VP4-SpyVLP (0.5 or 5 µg), SpyVLP (0.5 or 5 µg), 1-15 VP4-KLH (50 µg), 1-30 VP4-KLH (50 µg), 1-45 VP4-KLH (50 µg), or KLH (50 µg). All immunogens were adjuvanted with Magic Mouse Adjuvant, which contains immune-stimulatory CpG DNA oligodeoxynucleotides. Mice were boosted with the same dose of immunogen at 14 and 28 days. Tail bleeds were collected immediately prior to immunisation, 10 days post-immunisation, and 10 days post-boost 1. Terminal bleeds were collected 14 days post-boost 2 (Fig 3A). Each bleed was subsequently processed to separate the blood cells from the serum. Sera were then analysed by Virus Neutralisation Test (VNT) to assess the neutralising response to each immunogen. RV-A16 was incubated in the presence of endpoint sera at dilutions from 1 in 2 to 1 in 32, before infecting HeLa-H1 cells. These were used because they are known to be susceptible to RV-A16. Anti-VP4 neutralising control serum from a VP4-immunised sheep, which has previously been demonstrated to neutralise RV-A16 infections, was included as a positive control [20]. A no sera sample was also included as a negative control. Three days post-infection, cytopathic effect (CPE) was measured by crystal violet staining as a quantitative measure of cell viability [44]. Cells incubated in the presence of virus with no sera achieved complete cell death, giving an OD of 0.22. Cells incubated in the presence of anti-VP4 neutralising control sera showed no signs of CPE at a 1 in 2 or 1 in 4 dilution with OD readings between 1.2 and 2.8; CPE was visible at dilutions 1 in 8 and below, with an average OD of 0.51 at 1 in 8 and 0.24 at 1 in 16 OD (Fig 3B), indicating that the serum is fully neutralising at a 1 in 4 dilution and partially neutralising at a 1 in 8 dilution, which is consistent with previous studies [20].

Analysis of the mouse endpoint immune sera revealed that sera from immunisations with vector-only immunogens (SpyVLP-0.5, SpyVLP-5, or KLH, lacking a VP4 immunogen) had a mean OD of 0.27 to 0.35. This is close to the no-serum control reading of OD 0.22, which represents complete cell death. This indicates that mock sera do not protect cells from infection with RV-A16. However, a single serum sample in the SpyVLP-5 (vector only) and a single serum sample in the KLH (vector only) groups appeared to show low levels of non-specific neutralisation (Fig 3B). To aid visualisation of the data, we therefore set a cut-off (0.7 OD) for neutralisation for any values that were above this non-specific level. We are not able to explain the presence of low levels of non-specific neutralising antibodies in vector only immunised animals, but it was only observed in 2 out of a total of 15 vector-only control animals. It would have been interesting to analyse neutralisation in the pre-immune sera, but only the final bleed samples provided sufficient material for analysis by VNT.

For the VP4-containing immunogens, only the 1-15-VP4-SpyVLP-5 gave a neutralising response in all the mice in that group (Fig 3B). In the 1-15-VP4-SpyVLP-0.5 group, only 1/5 sera were neutralising, indicating that the 5 µg dose is required to generate a more consistent neutralising response. All 1-15-VP4-SpyVLP-5 sera were effective at neutralisation down to dilutions of 1 in 8.

The 1-15-VP4-KLH group had the second-highest number of neutralising sera, at 3/5. These sera were only capable of neutralising at a 1:2 dilution. In the remaining groups, the ability to neutralise was as follows, 2/5: 1-30-VP4-SpyVLP-0.5, 1/5: 1-30-VP4-SpyVLP-5, 2/5: 1-30-VP4-KLH, 1/5: 1-45-VP4-SpyVLP-0.5, 0/5: 1-45-VP4-SpyVLP-0.5 and 1/5: 1-45-VP4-KLH. No sera from these groups were neutralising below a 1 in 2 dilution.

In summary, among the immunogens tested, 1-15-VP4-SpyVLP-5 clearly produced the strongest and most reliable neutralising response. However, this level of neutralisation remains relatively low and a better understanding of the antigenicity of this immunogen to enhance the neutralising response would be a goal of further studies.

### Neutralising sera from mice immunised with 1-15-VP4 immunogens do not react with 1-45-VP4 peptides

Now that we had established that certain end-point sera were neutralising, we wanted to determine the overall strength of the peptide-specific immune response that developed during the immunisation experiment. For the initial investigation, we analysed sera from each bleed time point by ELISA using 1-45-VP4 peptide as the capture antigen (coated directly onto the plate). The complete immunogen was not used as a capture antigen to avoid detection of responses directed against the vector (SpyVLP or KLH). As all immunogens in this study contain a peptide sequence within the 1-45-VP4 peptide, we therefore expected the 1-45-VP4 peptide to capture all VP4-specific antibodies produced in this study.

**Fig 4:**
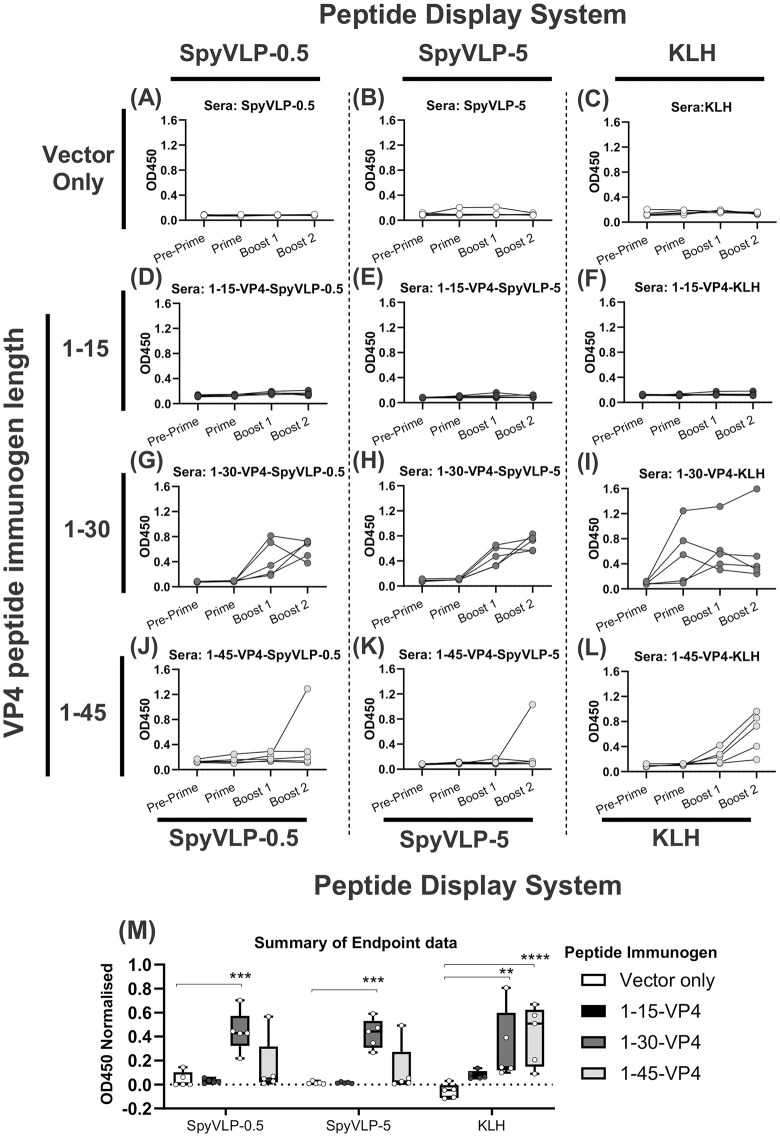
Neutralising sera from mice immunised with 1-15-VP4 immunogens do not react with 1-45-VP4 peptides. ELISAs using the VP4-1-45 peptide as a capture were used to assess the reactivity of immune sera. **(A-L)** Sera corresponding to pre-bleed, post-prime, post-boost 1 and post-boost 2 were used at a dilution of 1:160 in ELISA with VP4-1-45 peptide as the capture antigen. Sera from each group were used, SpyVLP-0.5 **(A)**, SpyVLP-5 **(B)**, KLH **(C)**, 1-15-VP4-SpyVLP-0.5 **(D)**, 1-15-VP4-SpyVLP-5 **(E)**, 1-15-VP4-KLH **(F)**, 1-30-VP4-SpyVLP-0.5 **(G)**, 1-30-VP4-SpyVLP-5 **(H)**, 1-30-VP4-KLH **(I)**, 1-45-VP4-SpyVLP-0.5 **(J)**, 1-45-VP4-SpyVLP-5 **(K)**, 1-45 VP4-KLH **(L)**. **(M)** Summary of endpoint (post boost 2) ELISA data in panels A-L. Data were normalised to positive and negative controls. Statistical analysis was performed to compare VP4-specific and vector-only sera using two-way ANOVA, and post-hoc analysis was performed using Sidak’s pairwise comparison with a significance threshold of p = 0.05. **p < 0.05, ***p < 0.005, ****p < 0.0005.

We found that sera from mice immunised with either SpyVLP-0.5, SpyVLP-5, KLH, 1-15-VP4-SpyVLP-0.5, 1-15-VP4-SpyVLP-5 or 1-15-VP4-KLH did not develop detectable reactivity with the 1-45-VP4 peptide (Fig 4A–4F, 4M). This is despite the observation that several sera from groups 1-15-VP4-SpyVLP-0.5, 1-15-VP4-SpyVLP-5 and 1-15-VP4-KLH neutralised RV-A16 in VNT (Fig 3B), indicating that they bind part of VP4 that is not accessible in the 1-45-VP4 peptide. This indicates the 1-45-VP4 peptide may adopt a different conformation to the 1–15 peptide or that the 1-45-VP4 peptide binds the plate in a way that obscures the 1–15 epitope.

Analysis of sera from 1-30-VP4-SpyVLP immunised mice revealed sera from 5/5 mice immunised with 1-30-VP4-SpyVLP-5 were reactive with VP4-1-45 peptide after the 1^st^ boost (Fig 4H). For 3/5 mice, a single boost was sufficient to reach maximum reactivity. For 2/5 mice, reactivity continued to increase after the 2^nd^ boost (Fig 4H). Similar results were observed for mice immunised with 1-30-VP4-SpyVLP-0.5 (Fig 4G). 5/5 mice in this group were reactive to the 1-45-VP4 peptide after the 1^st^ boost, while for 2/5 mice the reactivity continued to increase after the 2^nd^ boost (Fig 4G). This indicates that the 5 µg dose of 1-30-VP4-SpyVLP may initiate a slightly superior immune response after the 1^st^ boost.

After immunising with 1-30-VP4-KLH, we found that 5/5 mice had reactivity with the 1-45-VP4 peptide after the 1^st^ boost (Fig 4I). Sera from 1/5 mice had strong reactivity after the initial immunisation and it had much higher reactivity than all other mice. 4/5 had lower levels of reactivity than the mice immunised with 1-30-VP4-SpyVLP even after the 2^nd^ boost (Fig 4I). This indicates that presentation of the 1-30-VP4 peptide on the SpyVLP produces a superior immune response to the 1-30-VP4 peptide presented on KLH.

Sera from 1/5 mice immunised with 1-45-VP4-SpyVLP-0.5 and 1/5 mice immunised with 1-45-VP4-SpyVLP-5 were reactive with VP4 1-45 peptide; this reactivity only occurred after the 2^nd^ boost (Fig 4J, 4K).

Sera from 3/5 mice vaccinated with 1-45-VP4-KLH were reactive with the VP4-1-45 peptide after the 1^st^ boost. Reactivity increased after the 2^nd^ boost, where 4/5 mice sera were reactive (Fig 4L). This demonstrated that presentation of the VP4-1-45 peptide on KLH produces a superior immune response to presentation of VP4-1-45 peptide on the SpyVLP. Although 1-45-VP4-SpyVLP did generate an immune response, it was not consistent or reliable; this may relate to the high levels of aggregation observed in DLS (Fig 2B).

Given that sera from mice immunised with 1-15-VP4 immunogens (3/5: 1-15-VP4-KLH, 1/5: 1-15-VP4-SpyVLP-0.5 and 5/5: 1-15-VP4-SpyVLP-5) neutralised the virus in a VNT (Fig 3B), it is surprising that these sera had no detectable reactivity with the 1-45 peptide (Fig 4D–4F, 4M), even though they produced the strongest levels of neutralisation. 9/15 were neutralising at a 1 in 2 dilution, and 6/15 were neutralising at a 1 in 4 dilution (Fig 3B). All 15 immunisations with 1-30-VP4 immunogens produced sera that strongly reacted with 1–45 peptide (Fig 4G–4I, 4M), but only 5/15 were neutralising at a 1 in 2 dilution (Fig 3B). For the 1-45-VP4 immunogens, 7/15 reacted with the 1–45 peptide (Fig 4J–4L, 4M) and 2/15 were neutralising at a 1 in 2 dilution (Fig 3B). Therefore, neutralisation and reactivity with the 1–45 peptide do not correlate. This indicates the neutralising epitope may be poorly displayed in 1–45 peptides when presented on an ELISA plate.

**Fig 5:**
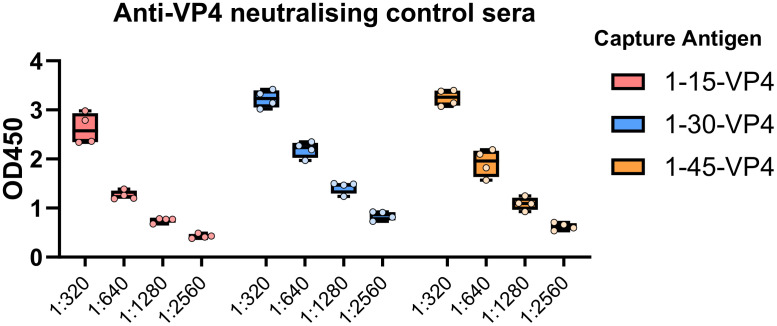
VP4 capture peptides are able to react with anti-VP4 positive control sera. Peptides VP4-1-15, VP4-1-30, and VP4-1-45 are functional as capture antigens in ELISA when used with anti-VP4 neutralising positive control sera. Wells were coated with capture peptides corresponding to either 1-15-VP4 (red), 1-30-VP4 (blue) or 1-45-VP4 (yellow). ELISAs were performed with a dilution range of 1:320 to 1:2,560. Each point corresponds to a technical replicate. Absorbance for all ELISAs was read at OD_450_. All ELISAs were performed using an anti-mouse IgG secondary antibody.

One possible explanation for why the neutralising sera raised against 1-15-VP4-KLH, 1-15-VP4-SpyVLP-0.5, or 1-15-VP4-SpyVLP-5 did not react with the 1-45 peptide is that peptides bind to the ELISA plate in a way that prevents interactions with the neutralising antibodies. For example, residues 1–15 of VP4 may preferentially bind to the plastic, meaning these residues are not accessible for antibodies to bind. To test this, we assessed the ability of the anti-VP4 neutralising control sera to bind peptides of different lengths (1-15-VP4, 1-30-VP4, and 1-45-VP4) coated directly onto ELISA plates (Fig 5). The anti-VP4 neutralising control sera readily reacted with peptides 1-15-VP4, 1-30-VP4, and 1-45-VP4, indicating that residues 1-15 of VP4 are accessible in peptides bound directly to ELISA plates (Fig 5).

### Peptide length and antigen display system influence the site of binding of VP4-specific antibodies

Since the neutralising sera raised against 1-15-VP4-KLH, 1-15-VP4-SpyVLP-0.5 or 1-15-VP4-SpyVLP-5 did not bind the 1–45 peptide, we hypothesised that the longer peptide may adopt a different conformation that influences the binding of VP4 antibodies. To determine whether the length of the VP4 peptide influences binding, we sought to identify the epitopes on VP4 that each serum recognised. This was carried out by two different ELISAs with peptides directly coated onto the plate. Firstly, we assessed the ability of sera to react with equimolar concentrations of all three peptide lengths used in the study (1-15-VP4, 1-30-VP4, 1-45-VP4) using a dilution range of 1 in 80 to 1 in 640. In addition, we mapped the position(s) on VP4 to which the sera bound, using a scanning library of 15-mer peptides with 5-amino-acid overlaps based on the first 55 N-terminal residues of RV-A16 VP4 and a sera dilution of 1 in 160 (Table 1). Together, these experiments allow us to evaluate how peptide length influences antigenicity.

**Fig 6:**
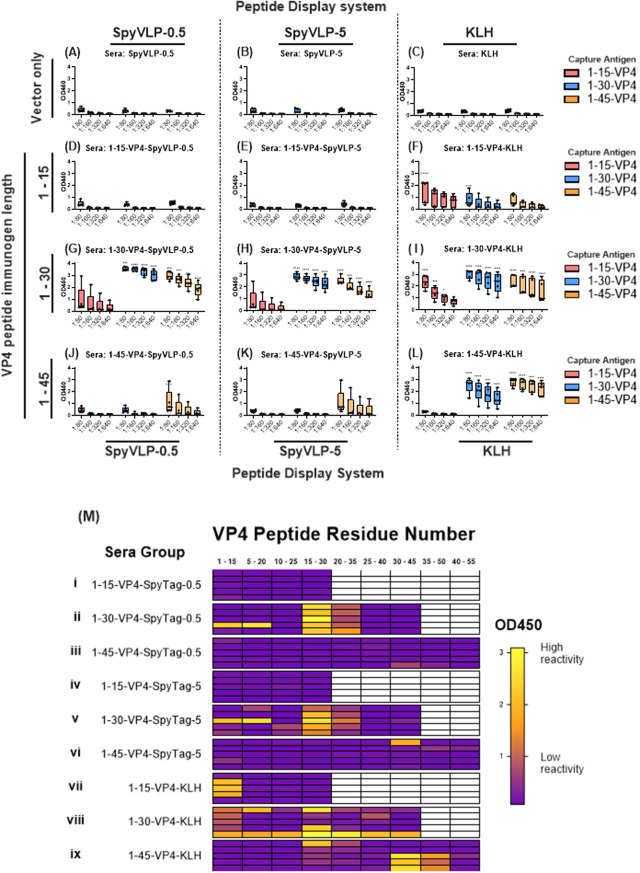
Peptide length and antigen display system influence the site of binding of VP4-specific antibodies. **(A-L)** ELISA was used to determine the reactivity of sera with capture antigens of different peptide lengths. Wells were coated with capture peptides corresponding to either 1-15-VP4 (red), 1-30-VP4 (blue) or 1-45-VP4 (yellow). ELISAs were performed for each serum group against each capture peptide using a dilution range of 1:80 to 1:640. Sera groups included SpyVLP-0.5 **(A)**, SpyVLP-5 **(B)**, KLH **(C)**, 1-15-VP4-SpyVLP-0.5 **(D)**, 1-15-VP4-SpyVLP-5 **(E)**, 1-15-VP4-KLH **(F)**, 1-30-VP4-SpyVLP-0.5 **(G)**, 1-30-VP4-SpyVLP-5 **(H)**, 1-30-VP4-KLH **(I)**, 1-45-VP4-SpyVLP-0.5 **(J)**, 1-45-VP4-SpyVLP-5 **(K)**, 1-45-VP4-KLH **(L)**. Absorbance was read at OD_450_. All ELISAs were performed using an anti-mouse IgG secondary antibody. Each point corresponds to a mouse (n = 5 per vaccination group). Data are shown as mean ± SD. Statistical analysis was performed using one-way ANOVA, and post-hoc analysis was performed using Dunnett’s test using the corresponding mock vaccination as the control mean. Significance threshold was set at p = 0.05. *p < 0.05, ***p < 0.0005, ****p < 0.00005. **(M)** Heat map visualisation of epitope mapping. Each individual serum was analysed by ELISA at a 1:160 dilution using a scanning library of 15-mer peptides with 5-amino-acid overlaps, based on the first 55 N-terminal residues of RV-A16 VP4. Scanning peptides acted as the capture antigen. Amino acid residue numbers of the capture peptides are displayed above each column. The sera group is labelled on the left: 1-15-VP4-SpyVLP-0.5 **(i)**, 1-30 VP4-SpyVLP-0.5 **(ii)**, 1-45-VP4-SpyVLP-0.5 **(iii)**, 1-15-VP4-SpyVLP-5 **(iv)**, 1-30-VP4-SpyVLP-5 **(v)**, 1-45-VP4-SpyVLP-5 **(vi)**, 1-15-VP4-KLH **(vii)**, 1-30-VP4-KLH **(viii)**, 1-45-VP4-KLH **(ix)**. Absorbance was read at OD_450_. ELISAs were performed using an anti-mouse IgG secondary antibody. Each row corresponds to a single mouse (n = 5 per immunisation group).

For the mock immunisation groups SpyVLP-0.5, SpyVLP-5 and KLH, no sera from immunisations with these immunogens reacted with any length of VP4 peptide (Fig 6A–6C). Analysis of the response against 1-15-VP4 peptide displayed by SpyVLP (1-15-VP4-SpyVLP) revealed that these sera did not react with any length of VP4 peptide, meaning that this immunogen did not induce an immune response that targets an epitope on free VP4 peptides (Fig 6D, 6E, 6M, 6M-i, 6M-iv). Given that the 1-15-VP4-SpyVLP-5 group was the most neutralising and the immunogen contained the 1-15-VP4 peptide, it is highly surprising that this serum has no detectable reactivity with the free 1-15-VP4 peptide.

For 1-15-VP4-KLH, 3/5 of the sera did react with 1-15-VP4, 1-30-VP4 and 1-45-VP4 peptides (Fig 6F). However, the reactivity was highest with the 1-15-VP4 peptide (Fig 6F). Given that 1–30 and 1–45 peptides contain the equivalent sequence of the 1-15-VP4 peptide, these findings indicate that residues 1–15 may be presented in a different conformation in the longer peptides, which reduces reactivity with the sera. This conformational change will likely either mask the epitope through steric hindrance, thereby making it less accessible to the sera, or change the epitope’s conformation so that the sera no longer recognise the peptide as efficiently. Further analysis by peptide scanning revealed that reactive 1-15-VP4-KLH sera only bind peptides that mapped to residues 1-15 (Fig 6M-vii). They did not react with peptides that mapped to residues 5–20 and above. This indicates that binding to the first 5 amino acids of VP4 is important for the reactivity of these sera.

Analysis of the 1-30-VP4-SpyVLP sera revealed that 1/5 of the sera from both the 0.5 µg and 5 µg groups reacted with the 1-15-VP4 peptide (Fig 6G, 6H, 6M-ii, 6M-v). 5/5 in both groups reacted with 1-30 and 1-45 peptides, which indicates that the major epitope for 1-30-VP4-SpyVLP is located between residues 15–30 (Fig 6G, 6H). Although reactivity was high with both the 1-30-VP4 and 1-45-VP4 peptides, reactivity was higher with the 1-30-VP4 peptide, indicating that this epitope may undergo a minor conformational change when the peptide length is increased from 30 to 45 amino acids (Fig 6G, 6H). Further analysis by peptide scanning confirmed that the dominant epitope was located between residues 15–30 (Fig 6M-ii, 6M-v). Although the highest level of binding mapped to residues 15–30, these sera also mapped to residues 20–35, but the signal was lower. 2/5 of the sera from 1-30-VP4-SpyVLP-5 also showed reactivity with 10–25, but at very low levels (Fig 6M-v); this reactivity was not observed in any 1-30-VP4-SpyVLP-0.5 sera (Fig 6M-ii). This region appears to form a minor epitope. Peptide scanning also revealed that sera from 1/5 mice from both the 1-30-VP4-SpyVLP-0.5 and 1-30-VP4-SpyVLP-5 groups were reactive with peptides 1–15 and 5–20, but not 10–25 (Fig 6M-ii, 6M-v). The signal was higher for the 5–20 peptide, indicating that this epitope is likely located between residues 5–15. Both sera that bound the 5–15 epitope also bound the dominant epitope at 15–30 described above (Fig 6M-ii, 6M-v). It is therefore not possible to distinguish between the signals from the two epitopes in ELISAs using the longer peptides. Consequently, we are unable to confirm if the 5–15 epitope is affected by increases in peptide length (Fig 6G, 6H). However, since binding to the 5–15 epitope was only observed in 1/5 mice for each of the 1–30-VP4-SpyVLP groups, these results indicate that 5–15 is less dominant than the 15–30 epitope, and it is therefore likely that the length of the peptide does affect the exposure of this epitope.

Different results were observed in the 1-30-VP4-KLH sera. 1/5 of the sera were reactive with all peptides used in the peptide scan (Fig 6M-viii). This included peptide 30-45, which does not contain any VP4 residues present in the 1-30-VP4 immunogen. It does however contain the 6 × lysines which are present in all peptides, this indicates that it does not bind a VP4 specific epitope and likely binds the lysines. This was the same serum that produced very high levels of 1-45-VP4 reactivity after the first immunisation (Fig 4I). Since this happened in only 1 out of the 60 mice immunised as part of this study, it means this is a rare occurrence, but this reinforced the benefit of including the lysine-containing sequence in the peptide scanning analysis. The remaining 4 sera all reacted with peptides 1-15-VP4, 1-30-VP4 and 1-45-VP4, with peptide 1-30-VP4 showing the highest level of reactivity (Fig 6I). These data would be consistent with a major epitope located somewhere in residues 1–15, with the conformation of this epitope most dominant in the 1–30 peptide. This was supported by peptide scanning results: 4/4 sera reacted with peptides mapping to residues 1–15, and 1/4 also bound peptides mapping to residues 5–20 (Fig 6M-viii). 2/4 sera could also bind peptides that mapped to residues 15–30, but, unlike the 1-30-VP4-SpyVLP, no strong reaction was observed with peptide 20–35. There was also a weak reaction with peptides 25–40 in an individual serum. These data indicate that, unlike in 1-30-VP4-SpyVLP, for 1-30-VP4-KLH, the dominant epitope may be within residues 1–15, and residues 15–30 only form a minor epitope. These data indicate that the conformation of 1–30 is altered, depending on whether 1–30 is conjugated to either SpyVLP or KLH. Alternatively, it could suggest that myristoylation influences antigenicity.

Analysis of the 1-45-VP4-SpyVLP sera revealed that only 1/5 of the sera from both the 5 µg and 0.5 µg groups were strongly reactive with the 1-45-VP4 peptide and there was little reactivity in any sera with 1-15-VP4 or 1-30-VP4 peptides (Fig 6J, 6K). This may indicate the presence of an epitope between residues 30–45. This was supported by peptide scanning, which revealed that the same sera bound peptides that mapped to residues 30–45 (Fig 6M-iii, 6M-vi). No reactivity was observed with closest overlapping peptides 25–40 or 35–50, indicating that the epitope is entirely located within residues 30–35 (Fig 6M-iii, 6M-vi). Also, the reactivity of the single positive 1-45-VP4-SpyVLP-0.5 serum was less reactive with the 30–45 peptide (Fig 6M-iii) than the 1–45 length peptide (Fig 6J), suggesting that the conformation of this epitope may not be optimal in the 30–45 peptide used in the peptide scanning. Given that this reactivity was only observed in a minority of the sera, the epitope in this 30–45 region may be poorly displayed.

For 1-45-VP4-KLH sera, all sera were reactive with peptides 1-30-VP4 and 1-45-VP4, but the reactivity was strongest with the 1-45-VP4 peptide, while no reactivity was observed for the 1–15 peptide (Fig 6L). There are two scenarios consistent with this result: firstly, that the sera could predominantly bind an epitope between residues 15–30, but the conformation is altered to become less dominant in the 1–30 peptide. Alternatively, the sera may bind multiple epitopes, one in 15–30 and another in 30–45. Peptide scans provided some clarification: although 4/5 sera showed high reactivity to the 1–30 peptide (Fig 6L), only 1 had a strong signal with the 15–30 peptide (Fig 6M-ix). This suggests that the sera that have high binding to the 1–30 peptide but lower levels of binding to the 15–30 peptide bind their target epitope in an area where the conformation is altered in the longer peptide. This conformation may be recapitulated poorly in the 15-mer peptides used in the scan. The peptide scan also showed that 3/5 of these sera reacted with peptides that mapped to 30–45 and 35–50, but not 25–40, indicating that these sera bind an epitope that spans residues 35–45 (Fig 6M-ix). 1/5 of the 1-45-VP4-KLH sera had no strong reactivity with any peptides in the scan, despite showing strong reactivity with 1–45 and weak reactivity with 1–30 (Fig 6L, Fig 6M-ix). This peptide did have relatively weak levels of reactivity with peptide 15–30 (Fig 6M-ix), indicating that the sera binds the full-length peptide in a way that is dependent on the conformation of the full-length peptide and this conformation is recapitulated poorly in the 15-mer peptides used in the scanning.

Overall, these data indicate that the structure of VP4 is highly variable and the conformation of the epitopes that are displayed is strongly dependent on the length of peptide and the display system used. The antigenic conformation of these epitopes also appears to vary between free peptides, peptides conjugated to SpyVLP, and peptides conjugated to KLH. Together, these factors appear to influence which part of VP4 the immune system targets. However, at present, we are unable to identify where or what the neutralising sera from 1-15-VP4-SpyVLP-immunised mice bind. Given that these sera neutralise the virus, they must bind the capsid. There are two potential explanations for these observations. The first is that the neutralising antibodies present in the sera could be a non-IgG isotype. All ELISAs up to this point used an anti-mouse IgG as a secondary antibody. If neutralising antibodies belong to a different isotype, such as IgM or IgA, our ELISAs would not be capable of detecting them. Alternatively, conjugation of the 1-15-VP4 peptide to the SpyVLP vector causes 1–15 to adopt a vastly different conformation than the free 1-15-VP4 peptide.

### Lack of peptide reactivity in 1-15-VP4-SpyVLP neutralising sera is not due to a mismatched isotype of detection antibody

**Fig 7:**
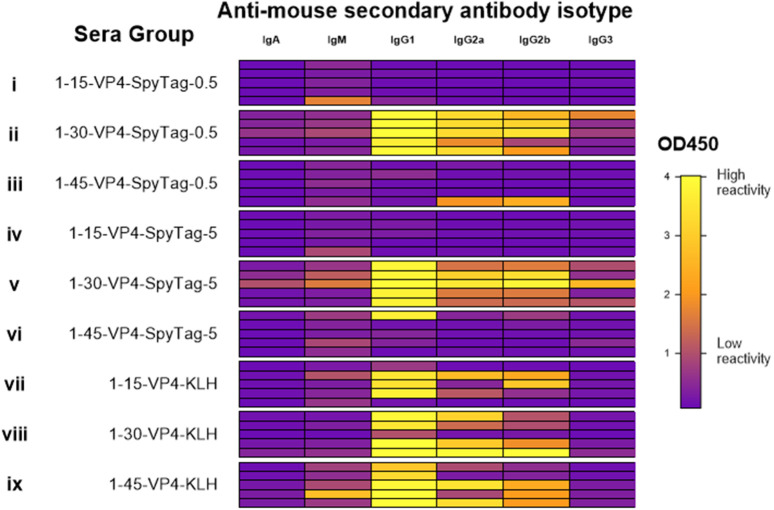
Lack of peptide reactivity in 1-15-VP4-SpyVLP neutralising sera is not due to a mismatched isotype of detection antibody. ELISAs were performed for each serum group with different isotypes of mouse secondary antibodies (IgA, IgM, IgG1, IgG2a, IgG2b, IgG3). Peptide 1-15-VP4 was used as a capture antigen for all sera raised against 1-15-VP4 peptides. Peptide 1-30-VP4 was used as a capture antigen for all sera raised against 1-30-VP4 peptides. Peptide 1-45-VP4 was used as the capture antigen for all sera raised against the 1-45-VP4 peptide. ELISAs were performed for each sera group at a 1:160 dilution, sera group is labelled on the left, 1-15-VP4-SpyVLP-0.5 **(i)**, 1-30 VP4-SpyVLP-0.5 **(ii)**, 1-45 VP4-SpyVLP-0.5 **(iii)**, 1-15-VP4-SpyVLP-5 **(iv)**, 1-30 VP4-SpyVLP-5 **(v)**, 1-45 VP4-SpyVLP-5 **(vi)**, 1-15 VP4-KLH **(vii)**, 1-30 VP4-KLH **(viii)**, 1-45 VP4-KLH **(ix)**. Absorbance was read at OD450. Each row corresponds to a single mouse (n = 5 per vaccination group). Each column corresponds to a different secondary antibody isotype.

All ELISAs up to this point used anti-IgG as the secondary antibody, and we hypothesised that the lack of detectable reactivity between neutralising sera from 1-15-VP4-SpyVLP-immunised mice and free VP4 peptide may be due to the neutralising sera containing non-IgG isotype antibodies. To determine the isotypes of VP4-specific antibodies in each serum, we screened the sera in ELISA using their cognate peptide as the capture, i.e., peptide 1-15-VP4 was used as capture antigen for all sera raised against 1-15-VP4 peptides, peptide 1-30-VP4 was used as capture antigen for all sera raised against 1-30-VP4 peptides and peptide 1-45-VP4 was used as capture antigen for all sera raised against 1-45-VP4 peptides. We then screened the sera against a panel of different secondary antibodies, including anti-mouse IgA, IgM, IgG1, IgG2a, IgG2b or IgG3 (Fig 7). In these experiments, we observed significant reactivity with antibodies that detect IgG isotypes, or low-level reactivity with antibodies that detect IgM isotypes. The results were consistent with existing data generated using the standard anti-IgG secondary (which can recognise all mouse IgG sub isotypes, including IgG1, IgG2a, IgG2b and IgG3 antibodies). For example, sera from 1-15-VP4-SpyVLP-0.5 and 1-15-VP4-SpyVLP-5 immunised mice, which were previously found not to be reactive with the standard anti-IgG secondary, were also not reactive with secondary antibodies against any specific IgG subtype (Fig 7-i, 7-iv). Antibodies previously shown to be reactive with a standard anti-IgG secondary were also highly reactive with at least one of the secondary antibodies against a specific IgG subtype. This includes sera from 1-30-VP4-SpyVLP-0.5, 1-45-VP4-SpyVLP-0.5, 1-30-VP4-SpyVLP-5, 1-45-VP4-SpyVLP-5, 1-15-VP4-KLH, 1-30-VP4-KLH, 1-45-VP4-KLH immunised mice (Fig 7-ii, 7-iii, 7-v, 7-vi, 7-vii, 7-viii, Fig 7-ix). Except for 1-45-VP4-SpyVLP-0.5 (Fig 7-iii), all these sera reacted most strongly with IgG1. This sample, which had previously shown reactivity using the pan-IgG secondary antibody, had no detectable IgG1 reactivity but was reactive with IgG2a and IgG2b. (Fig 7-iii).

Assessment of reactivity with an anti-mouse IgA secondary revealed that all sera had no or very little IgA (Fig 7).

Assessment of reactivity with an anti-mouse IgM secondary revealed that several sera, such as 1-30-VP4-SpyVLP-5 and 1-45-VP4-KLH, that had strong reactivity with anti-IgG secondaries also reacted with anti-IgM (Fig 7-v, 7-ix). A single serum from 1-15-VP4-SpyVLP-0.5 reacted with anti-IgM, but this serum was not neutralising (Figs 7-i, 3B). However, none of the neutralising 1-15-VP4-SpyVLP-5 sera or the single neutralising serum from 1-15-VP4-SpyVLP-0.5 showed any substantial reactivity with anti-IgM (Fig 7-iv, 7-i). Taken together, the lack of reactivity between the neutralising sera and VP4 peptides is not due to the neutralising sera belonging to a non-IgG isotype.

### Neutralising 1-15-VP4-SpyVLP sera only react with 1-15-VP4 peptide when conjugated to a display system

We would expect serum that neutralises RV-A16 to contain antibodies that bind the virus capsid. As described above, it was therefore surprising that neutralising sera from 1-15-VP4-SpyVLP-immunised mice do not react with peptides corresponding to the VP4 capsid protein. We hypothesised that the neutralising 1-15-VP4-SpyVLP sera bind a native ‘virus-like’ conformational epitope within the first 15 N-terminal amino acids of VP4. This epitope may be induced by conjugation of the peptide to SpyVLP but would be poorly recapitulated in the free peptide.

**Fig 8:**
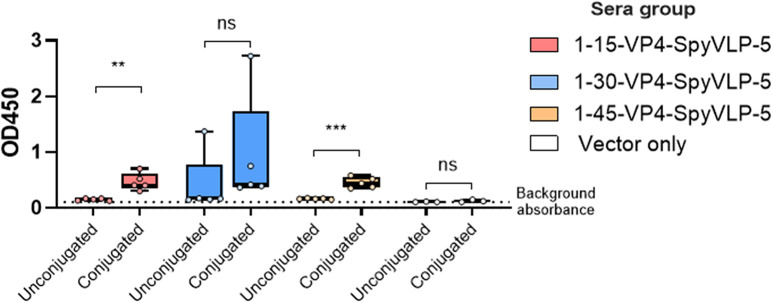
Neutralising 1-15-VP4-SpyVLP sera only react with 1-15-VP4 peptide when conjugated to a display system. ELISAs were performed to assess if conjugation of 1-15-VP4 peptide to DogCatcher-MBP affected its reactivity with sera from 1-15-VP4-SpyVLP-5, 1-30-VP4-SpyVLP-5 or 1-45-VP4-SpyVLP-5 immunised mice. Wells were coated with equimolar concentrations of either unconjugated or conjugated 1-15-VP4 solution to act as capture antigens. ELISAs were carried out using a 1:160 dilution with 1-15-VP4-SpyVLP-5 (red), 1-30-VP4-SpyVLP-5 (blue) or 1-45-VP4-SpyVLP-5 (yellow) or vector only as a negative control (white). An IgG secondary antibody was used. Background absorbance is represented by a black dashed line. Absorbance was read at OD_450_. Each point corresponds to a mouse (n = 5 per vaccination group). Data is shown as mean ± SD. Statistical analysis was performed using one-way ANOVA, and post-hoc analysis was performed using Dunnett’s test using the corresponding mock vaccination as the control mean. Significance threshold was set at p = 0.05. *p < 0.05, ***p < 0.0005.

As expected, sera from mice immunised with SpyVLP peptide conjugates (1-15-VP4-SpyVLP, 1-30-VP4-SpyVLP, 1-45-VP4-SpyVLP) had high levels of reactivity with SpyVLP. We were therefore unable to assess the peptide-specific response using the peptide-SpyVLP immunogen as a capture. To overcome this issue, we tested whether conjugation of VP4-1-15 peptide to an alternative peptide display system would reveal any peptide reactivity in the sera. For this, we used an orthogonal peptide/protein pair that forms a spontaneous isopeptide bond, DogTag:DogCatcher [45], to conjugate VP4-1-15 peptide to a maltose-binding protein (MBP) fused to DogCatcher. A subset of serum groups was selected (1-15-VP4-SpyVLP-5, 1-30-VP4-SpyVLP-5, 1-45-VP4-SpyVLP-5) to represent both neutralising and non-neutralising sera. ELISA plates were coated with either conjugated 1-15-VP4-DogTag:DogCatcher-MBP or an unconjugated mix of DogCatcher-MBP and 1-15-VP4-SpyTag (peptide tagged with SpyTag; not compatible for conjugation to DogCatcher). Wells were then probed with sera from 1-15-VP4-SpyVLP-5, 1-30-VP4-SpyVLP-5 or 1-45-VP4-SpyVLP-5 immunised mice. As expected, the single 1-30-VP4-SpyVLP-5 sera that previously reacted with free 1-15 peptide (Fig 6H, 6M-v) also reacted with the unconjugated 1-15-VP4 and DogCatcher-MBP (Fig 8). For the remaining four 1-30-VP4-SpyVLP sera, 5/5 of the 1-15-VP4-SpyVLP-5 sera, and 5/5 of the 1-45-VP4-SpyVLP-5 sera, reactivity with the unconjugated 1-15-VP4 and DogCatcher-MBP was at background levels (Fig 8). This demonstrates that these sera were unreactive with both the free 1-15-VP4 peptide or DogCatcher-MBP. However, conjugation of 1-15-VP4-DogTag to DogCatcher-MBP enhanced reactivity for all sera that were non-reactive with the unconjugated mix of 1-15-VP4 and DogCatcher-MBP (Fig 8). This indicates that there are antibodies present in 1-15-VP4-SpyVLP-5, 1-30-VP4-SpyVLP-5, and 1-45-VP4-SpyVLP-5 sera that are only reactive with the 1-15 peptide when it is conjugated to a display system and are not reactive with the free peptide. These sera likely react with a conformational epitope in 1-15-VP4 that only becomes accessible upon conjugation of the 1-15-VP4 peptide to SpyVLP or DogCatcher-MBP peptide presentation systems. However, given that this was observed in both neutralising and non-neutralising sera, this observation does not explain why some sera are neutralising while others are not.

### Structural analyses indicate that residues 1-15 adopt different conformations in 1-15-VP4, 1-30-VP4 and 1-45-VP4

Results from Fig 6 suggest that the antigenicity of the peptides used in the study varies with peptide length. We propose that this is due to the different peptides having alternative antigenic conformations. To test this hypothesis, we used AlphaFold 3 to predict structural models of peptides 1-15-VP4-SpyTag, 1-30-VP4-SpyTag, and 1-45-VP4-SpyTag. Sequences used for the predictions included the 6x Lysines and the SpyTag as shown in Table 1.

**Fig 9:**
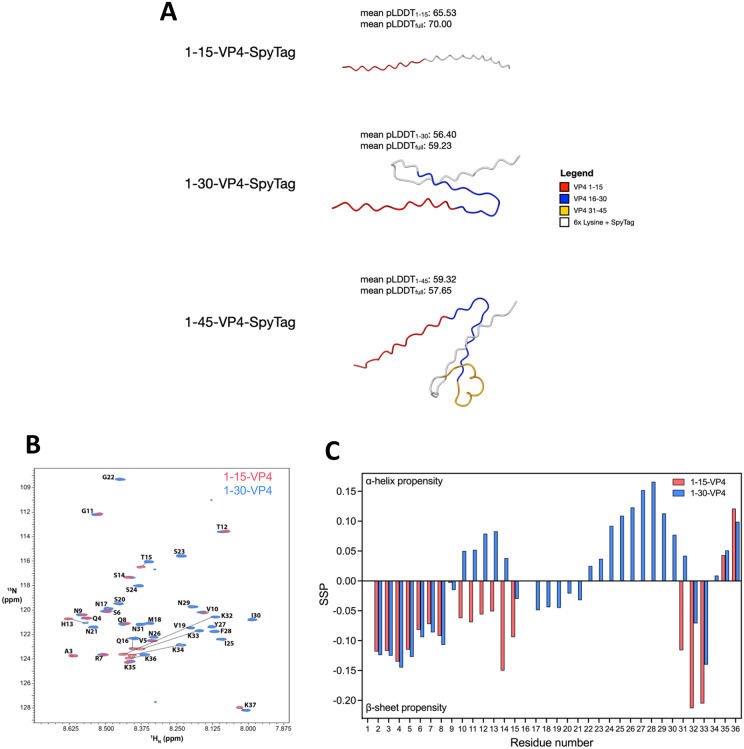
Structural analyses indicate that residues 1-15 adopt different conformations in 1-15-VP4, 1-30-VP4 and 1-45-VP4. **(A)** Cartoon representations of the structural models for peptide monomers predicted using AlphaFold 3. Residues corresponding to VP4 regions and tags are colour-coded as indicated in the legend. The mean pLDDT scores for VP4 residues only and for all residues are indicated above the models. **(B)** Spectral overlay of ^1^H,^15^N HMQCs for the backbone amides of 1-15-VP4 and 1-30-VP4 with cross peaks labelled with the corresponding residue. **(C)** Per-residue secondary structure propensities (SSP) for 1-15-VP4 and 1-30-VP4 peptides. Positive values indicate β-strand propensity and negative values indicate α-helical propensity. SSP values of +1 or -1 would correspond to the presence of a completely ordered β-strand or α-helix, respectively.

The predictions show that the peptides containing only residues 1–15 are linear and do not have regular secondary or higher order structure, while the inclusion of residues 16–30 or 16–45 results in the peptide folding into a more complex structure (Fig 9A). This increased secondary structure formation could potentially mask antigenically important sites in the longer peptides, the pLDDT values indicated low to moderate confidence for these models [46], which can also indicate structural dynamics [47].

The structure and dynamics of the 1-15-VP4 and 1-30-VP4 peptides were further investigated using nuclear magnetic resonance (NMR) spectroscopy. This analysis focused on peptides in solution; therefore, peptides comprised the VP4 sequence and 6 lysines, without the SpyTag sequence. The assigned backbone amide ^1^H and ^15^N chemical shifts indicated small but significant perturbations in residues 1–15 between the two peptides (Fig 9B). Peptide secondary structure propensities determined from the ^1^H_α_ and ^13^C_α_ chemical shifts [48] indicated that residues 10–14 adopted different conformations in 1-15-VP4 and 1-30-VP4. These residues were in a partially extended conformation in 1-15-VP4 but partially helical in 1-30-VP4 (Fig 9C). In addition, residues 17-21 of 1-30-VP4 exhibited β-sheet propensity. These results are consistent with the AlphaFold predictions, indicating that the additional residues of 1-30-VP4 allow the peptide to adopt an altered conformation with different antigenicity.

## Discussion

In the current study, we sought to identify conditions for creating an RV VP4 immunogen that elicits the greatest possible neutralising response. We compared three VP4 peptide lengths (1-15-VP4, 1-30-VP4, and 1-45-VP4) conjugated to SpyVLP. We included peptides presented on KLH as an established approach. We showed that the combination of the 1-15-VP4 peptide and SpyVLP display system (1-15-VP4-SpyVLP) produced the highest levels of neutralising antibodies. Other immunogens either elicited a response 8 times lower than that of 1-15-VP4-SpyVLP or induced no neutralising response at all. The 1-15-VP4-SpyVLP immunogen also produced the most consistent response: a 5µg dose of 1-15-VP4-SpyVLP produced a neutralising response in all 5 mice, while no other immunogen achieved this.

Mapping reactivity of the resulting sera to free peptides identified several antigenic sites in RV-A16 VP4, including sites that map to residues 1–15 and 15–30. However, antibody reactivity to these sites did not correlate with neutralisation. This was surprising given that previous studies with other picornaviruses identified neutralising antibodies that reacted with VP4 residues 7–15 in RV-B14 and residues 5–12 and 25–35 in EV-A71 [19,22,23]. Instead, in our study, VP4-specific antibody neutralisation of RV-A16 depended on binding to an epitope in 1–15-VP4. We propose that 1-15-VP4-SpyVLP produced the highest level of neutralising antibodies because it most accurately recapitulates a virus-like conformation of an epitope located in VP4 residues 1–15 and which is exposed at the virus surface during capsid breathing. Alternatively, it is still possible that antibodies targeting other sites may also be neutralising, but they did not achieve a high enough titre in this study, or that the aggregation observed with VLPs bound to longer peptides obscured neutralising epitopes.

Further detailed mapping of the different sera produced in this study revealed that the antigenic conformation of VP4 is highly variable and is influenced by peptide length and display system. This was supported by AlphaFold predictions and NMR data suggesting that the different lengths of peptides can have distinct structural conformations. Examples of peptide length influencing antigenic conformation are demonstrated by the different reactivities of some sera with peptides of different lengths. The 1-15-VP4-KLH sera reacted with free 1-15-VP4 peptide but did not react with equimolar concentrations of 1-30 and 1-45 peptides (Fig 6F). This indicates that residues 1–15 may be presented in a different conformation in the longer peptides, which reduces reactivity with the 1-15-VP4-KLH sera. Furthermore, 1-30-VP4-SpyVLP generates a strong response directed against residues 15–30 of VP4 (Fig 6G, 6H, 6M-ii, 6M-v). However, sera generated in response to the 1-45-VP4-SpyVLP, which also contains the 15–30 sequence, had no detectable response against peptides mapping to this region (Fig 6J, 6K, 6M-vi). This indicates that presentation of the 15–30 region of VP4 differs between 1-45-VP4-SpyVLP and 1-30-VP4-SpyVLP.

By comparing reactivities of the 1-30-VP4-SpyVLP and 1-30-VP4-KLH sera, we demonstrate that the display system also influences the antigenic conformation. Most sera from mice immunised with peptides presented on KLH (1-15-VP4-KLH or 1-30-VP4-KLH) were reactive with free 1-15-VP4 peptide. However, sera from mice immunised with peptides presented on SpyVLP (1-15-VP4-SpyVLP or 1-30-VP4-SpyVLP) were unreactive with free 1-15-VP4 peptide, but they were reactive with the peptide after conjugation to a DogCatcher-MBP display system. These data indicate that the 1-15-VP4 peptide may undergo a conformation change upon conjugation to the SpyVLP or DogCatcher-MBP but not KLH. The differences we detected here, however, do not explain the cause of the superior neutralisation ability of 1-15-VP4-SpyVLP sera, since non-neutralising sera from 1-30-VP4-SpyVLP and 1-45-VP4-SpyVLP produced similar levels of reactivity with the conjugated 1-15-VP4 antigen. We therefore suspect that there are further conformational differences between 1-15-VP4-SpyVLP and 1-30-VP4-SpyVLP/1-45-VP4-SpyVLP that we cannot detect in our ELISA.

Previous studies with other picornaviruses have also indicated that peptide length and the display system influence the antigenic conformation of VP4. Immunisations with RV-B14 peptides corresponding to VP4 1–24 generated neutralising antibodies reactive with free VP4 1–24 peptides and 15-mer peptides containing residues 7–15 of VP4. In contrast, immunisation with peptides corresponding to residues 1–30 generated neutralising antibodies that were reactive with full-length VP4 but had no detectable reactivity with the 1-24-VP4 peptide or any 15-mer peptides containing residues 7–15 [19]. This suggests that an increase in peptide length by just 6 amino acids is sufficient to alter the antigenicity of VP4. For EV-A71, mice were immunised with a Hepatitis B Virus core fusion protein VLP containing the N-terminal 20 amino acids of EV-A71 VP4 and this generated neutralising antibodies that bound the 5–12 region of VP4 [23]. However, in a separate study, a phage display screen using full-length VP4 as bait only generated antibodies that bound between residues 25–34 [22,23]. The phage display screen did not yield antibodies that bound the 5–12 epitope, indicating that the 5–12 epitope is not available in the full-length protein. These existing data are consistent with the data in our current study and support our proposal that antigenic conformation of VP4 is highly variable and impacts the induction of neutralising antibodies.

The ability of VP4 to adopt multiple conformations is likely related to the multiple biological functions required of this protein in capsid assembly and stability, uncoating, membrane interactions and host response suppression [26,30,49–51]. Structural studies of antibody-antigen complexes would facilitate a better understanding of peptide conformation and determine the optimal conformations for neutralisation.

Understanding the mechanism of neutralisation by VP4-specific antibodies would also be very interesting. Antibodies may bind to VP4 N-terminal epitopes during transient exposure of the VP4 N-terminus upon capsid breathing. This could neutralise virus by hindering receptor binding or the immune complex may still be able to bind to receptor and be internalised but with reduced ability to interact with and permeabilise the endocytosed vesicle.

While the current study has focused on neutralising antibody responses, it is possible that the immunogens tested in this study may produce protective T-cell responses or non-neutralising antibodies may initiate other modes of protection, such as antibody-dependent cellular phagocytosis (ADCP). Previous studies have shown that antibodies targeting the C-terminus of RV VP1, which, like VP4, is an internal epitope, are non-neutralising but can protect via ADCP [52]. In addition, antibodies targeting the C-terminus of VP4 may contribute to a protective T-cell response in mice [18,53].

In conclusion, this study has demonstrated that presentation of the 1–15 region of VP4 in a specific antigenic conformation is important for the generation of neutralising VP4 antibodies. However, given that immunogens containing the same region produce different antigenic responses to that region, we hypothesise that the antigenic conformation of VP4 varies greatly between peptides of different lengths and identical peptides presented on different display systems. Furthermore, even the best immunogen developed in this study produced relatively low levels of neutralising antibodies. This highlights the complexities of creating an effective response against VP4. Future efforts to develop a VP4 vaccine would need to focus on improving neutralising antibody titres, but also to investigate if non-neutralising VP4 responses contribute to protection against RV infection. This work could also impact the design of other peptide vaccines, since a highly variable antigenic conformation may not be unique to VP4 peptides and may be a common characteristic of other peptides.

## Materials and methods

### Ethical statement

Mouse experiments were performed according to the UK Animals (Scientific Procedures) Act, Project Licence (PP8335202) and approved by The Pirbright Institute Animal Request Review Body. All experiments were done in compliance with the ethical regulations for animal testing and research. All conditions were in accordance with or surpassed the UK Home Office ethical and welfare guidelines. Female BALB/c mice (~9 weeks old at the time of first immunisation) were obtained from Envigo (now Inotiv). Only female animals were used to minimise potential aggression within groups. Mice were housed in a large mouse breeding cage rather than an M1 mouse cage, and bedding was corn cob. Mice were provided with mouse swings and red plastic items to climb on/hide in as environmental enrichment (e.g., igloos and tubes). Mice were handled using clear tubes rather than tail handling. Mice were fed on RM1 (e) Maintenance diet with water *ad libitum*. The mouse room was maintained at 20.0–21.4 °C with 57.7%–61.6% humidity. The light cycle was 12 h on, 12 h off with a lux reading of at cage level of ~ 25 Lux.

### Mouse immunisations and sampling

To prepare the SpyVLPs for vaccination, 10 μM SpyTag-VP4 was conjugated with 10 μM of SpyCatcher003-mi3 in PBS at 25 °C for 18 h. The reaction was centrifuged for 30 min at 17,000 × g at 4 °C to remove potential aggregates. Doses were diluted to either 10 µg/ml (SpyVLP-VP4-0.5) or 100 µg/ml (SpyVLP-VP4-5) in sterile PBS. For Spy-VLP immunisations, fresh SpyCatcher-conjugated immunogens were prepared for each boost. For KLH immunisations, unfrozen immunogen was used for the first immunisation, and for the boosts, aliquots of immunogen that had undergone one freeze-thaw were used. KLH doses were diluted to 1,000 µg/ml in sterile PBS. Before subcutaneous (SC) immunisation, each sample was mixed 1:1 with MagicMouse (Gentaur or Invivogen) and immunised with 100 µL of this solution. This resulted in a final dose of either 0.5 µg for SpyVLP-0.5, 5 µg for SpyVLP-VP4-5, or 50 µg for KLH.

Mice were prime-boost-boost vaccinated subcutaneously with either 1-15-VP4-SpyVLP-0.5, 1-30-VP4-SpyVLP-0.5, 1-45-VP4-SpyVLP-0.5, SpyVLP-0.5, 1-15-VP4-SpyVLP-5, 1-30-VP4-SpyVLP-5, 1-45-VP4-SpyVLP-5, SpyVLP-5, 1-15-VP4-KLH, 1-30 VP4-KLH, 1-45 VP4-KLH or KLH. Each vaccination group consisted of 5 mice. Bleeds were taken from the tail 10 days and 24 days post-immunisation. At day 42, mice were anaesthetised and terminated by cardiac puncture terminal bleeding. The anaesthetic used was a cocktail of 300 mg/kg Ketamine and 3 mg/kg Medetomidine dosage, according to mouse weight. To prepare the anaesthetic cocktail, equal volumes of 100 mg/mL Ketamine and 1 mg/mL Medetomidine were mixed thoroughly. Volume administered to each mouse was based on their weight; e.g., for a 25 g mouse, 0.15 mL of this cocktail was administered. The ketamine/medetomidine anaesthetic ensured that mice did not feel the cardiac puncture and that they had a high heart rate. This enables a higher volume of blood to be harvested from each mouse. Mice were confirmed to be unresponsive prior to cardiac puncture. Bloods were collected into 1.5 mL microcentrifuge tubes and allowed to clot at 22 °C for 1 h, then centrifuged at 10,000 × g for 5 min at 22 °C. The clarified sera were stored at -20 °C.

The number of animals used was intended to be the minimum number necessary to generate robust data and achieve the experiment’s scientific objectives. Group sizes of 5 were based on variation in antibody titres seen in previous studies. Power calculations showed that such a group size is likely to produce statistically relevant data. For example, immunising mice with picornavirus-derived peptides, we would expect a 4 log2 difference in virus-specific antibody titres with a standard deviation of 1.4 log2. A group size of five mice will detect differences of these magnitudes and with these standard deviations with 90% power and 95% confidence (computed using the power.t.test function in R).

### Peptides

RV-A16 VP4 peptides (Table 1) were synthesised by Peptide Synthetics; peptides were supplied at >95% purity validated by HPLC-MS. Peptides were dissolved at 33.3 mM in dimethyl sulfoxide (D2650, Sigma). Stocks were then aliquoted and stored at -20 °C until further use. Peptides were either conjugated to display vectors to create immunogens or used as capture antigens in ELISAs. Peptides used as capture antigens for ELISAs were diluted in 0.05 M carbonate-bicarbonate buffer (3.7 g NaHCO_3_, 0.64 g Na_2_CO_3_, in 1 L of distilled water and pH adjusted to 9.6) to a concentration of 2.4 mM.

### Expression and purification of SpyCatcher003-mi3 and DogCatcher-MBP display systems

pET28a-SpyCatcher003-mi3 (GenBank MT945417, Addgene 159995) [40] and pET28a-AviTag-DogCatcher-MBP (GenBank MZ365293, Addgene 171928) [45] were transformed into *E. coli* BL21(DE3)-RIPL cells. They were grown on LB-Agar plates with 50 μg/mL kanamycin for 16 h at 37 °C. A single colony was used to inoculate 10 mL LB medium containing 50 μg/mL kanamycin and grown for 16 h at 37 °C with shaking at 200 rpm. This culture was added to 1 L LB + 0.8% (w/v) glucose containing 50 μg/mL kanamycin and incubated at 37 °C and 200 rpm shaking until OD_600_ reached 0.6. Cultures were induced with 0.5 mM isopropyl β-D-1-thiogalactopyranoside (IPTG). For SpyCatcher003-mi3, cells were grown at 22 °C with shaking at 200 rpm for 16 h. For AviTag-DogCatcher-MBP, cells were grown at 30 °C with shaking at 200 rpm for 4 h. Cultures were pelleted by centrifugation at 4,000 x g.

AviTag-DogCatcher-MBP pellets were resuspended in 20 mL Ni-NTA buffer (50 mM Tris-HCl pH 7.8 containing 300 mM NaCl), and SpyCatcher003-mi3 pellets were resuspended in 20 mL 20 mM Tris-HCl, 300 mM NaCl, pH 7.9 at 25 °C. In both cases, the pellets were supplemented with 0.1 mg/mL lysozyme, 1 mg/mL complete mini EDTA-free protease inhibitor (Roche), and 1 mM phenylmethanesulfonyl fluoride (PMSF) and incubated for 30 min. An Ultrasonic Processor equipped with a microtip (Cole-Parmer) was used to sonicate on ice (4 times for 60 s, 50% duty-cycle). Cell debris was cleared by centrifugation at 35,000 x g for 45 min at 4 °C.

AviTag-DogCatcher-MBP was purified by nickel-nitrilotriacetic acid (Ni-NTA) affinity chromatography. Ni-NTA agarose (Qiagen) was packed in an Econo-Pac Chromatography Column (Bio-Rad). It was washed with 2 × 10 CV of Ni-NTA buffer. The bacterial supernatant was incubated in the Ni-NTA column for 1 h at 4 °C with rolling. The supernatant was allowed to flow through by gravity before being washed with 2 × 10 CV of Ni-NTA wash buffer (10 mM imidazole in Ni-NTA buffer). The resin was incubated with Ni-NTA elution buffer (200 mM imidazole in Ni-NTA buffer) for 5 min before eluting by gravity. A total of six 1 CV elutions were performed. Elution fractions were assessed by SDS-PAGE with Coomassie staining, pooled, and dialysed for 16 h against a 1,000-fold excess of PBS before being stored at -80 °C.

SpyCatcher003-mi3 was purified by ammonium sulfate precipitation. 170 mg of ammonium sulfate was added per mL of cleared lysate. The mixture was incubated at 4 °C for 1 h with stirring at 100 rpm to precipitate the particles. The solution was centrifuged for 30 min at 30,000 x g at 4 °C, and the pellet was resuspended in 10 mL mi3 buffer (25 mM Tris–HCl, 150 mM NaCl, pH 8.0) at 4 °C. The resuspended pellet was filtered sequentially through 0.45 µm and 0.22 µm syringe filters (Starlab).

The filtrate was dialysed for 16 h against a 1,000-fold excess of mi3 buffer using a 100 kDa molecular weight cutoff Spectra/Por-3 dialysis membrane (Spectrum Labs). The dialysed particles were centrifuged at 17,000 x g for 30 min at 4 °C and filtered through a 0.22 µm syringe filter. The filtrate was loaded onto a HiPrep Sephacryl S-400 HR 16–600 column (GE Healthcare), which had been equilibrated with filtered mi3 buffer using an ÄKTA Pure 25 system (GE Healthcare). The protein was run over the column at 0.1 mL/min while collecting 1 mL elution fractions. The fractions containing the purified SpyCatcher003-mi3 were pooled and concentrated using a Vivaspin 20 100 kDa molecular weight cut-off centrifugal concentrator (GE Healthcare).

SpyCatcher003-mi3 was endotoxin-depleted using Triton X-114 phase separation. For this procedure, all microcentrifuge tubes were pyrogen-free, and all pipette tips were filtered. 1% (v/v) Triton X-114 was added to SpyCatcher003-mi3 samples, and the solution was mixed by gentle pipetting. The samples were incubated on ice for 5 min. The solution was transferred to a ThermoMixer (Eppendorf) at 37 °C, incubated for 5 min, and centrifuged for 1 min at 16,000 x g at 37 °C. The top phase was transferred into a fresh tube. This procedure was repeated a total of three times. After the third round, an additional incubation at 37 °C for 5 min, followed by centrifugation at 16,900 x g for 2 min at 37 °C, was performed to remove residual Triton X-114. The endotoxin content of all recombinant antigens was measured using the Limulus amoebocyte lysate (LAL)-based Chromogenic Endotoxin Quantitation Kit (Thermo Fisher) following the manufacturer’s instructions. All particles were below the acceptable levels of 20 EU/mL [54]. The concentration of endotoxin-depleted particles was measured using bicinchoninic acid (BCA) assay (Pierce) and then stored at -80 °C.

### Conjugation of VP4 peptides to display systems

VP4 peptides 1-15-VP4-SpyTag, 1-30-VP4-SpyTag or 1-45-VP4-SpyTag at 10 µM were conjugated to SpyVLP at 10 µM in PBS at 25 °C for 18 h. Possible aggregates were then removed by centrifugation at 16,900 g for 30 min at 4 °C.

1-15-VP4-DogTag at 10 µM was conjugated to DogMBP at 10 µM in PBS at 25 °C for 2 h.

Conjugation of KLH to myristoylated 1-15-VP4, 1-30-VP4, and 1-45-VP4 peptides was performed by Peptide Synthetics. KLH conjugation works by conjugation to a reactive Cys group on the peptide on the most C-terminal residue.

### SDS-PAGE

Samples were mixed with reducing 6 × loading dye (0.23 M Tris–HCl, pH 6.8, 24% (v/v) glycerol, 120 μM bromophenol blue, 0.23 M SDS, 0.2 M dithiothreitol) and resolved on 12% SDS–PAGE. Gels were then stained with InstantBlue Coomassie (Expedion) and imaged using a ChemiDoc XRS imager (Bio-Rad).

### DLS

Samples were centrifuged for 30 min at 16,900 × g at 4 °C to pellet possible aggregates. Before each measurement, the quartz cuvette was incubated in the instrument for 5 min to stabilise the sample temperature. Samples were measured at 100 μg/mL SpyVLP. 30 μL of sample was measured at 20 °C using an OmniSIZE (Viscotek) with 20 scans of 10 s each. The settings were 50% laser intensity, 15% maximum baseline drift, and 20% spike tolerance.

### Cells and virus

H1 HeLa cells (CRL-1958, ATCC) were propagated in 10% (v/v) foetal bovine serum (FBS) (26140087, Gibco) + 1% (v/v) penicillin-streptomycin (P/S) (15140122, Gibco) in Dulbecco’s Modified Eagle Medium (DMEM) (41965039, Gibco) at 37 °C and 5% (v/v) CO_2_. 80–90% confluent cells were split by washing with sterile PBS and incubation with 0.25% (v/v) trypsin-EDTA (25200056, Gibco) for 3 min. Trypsin was inactivated with 10% (v/v) FBS-DMEM, and cells were pelleted at 900 x g for 5 min at 22 °C. Supernatants were discarded, and pellets were resuspended and diluted in 10% (v/v) DMEM for use in subsequent experiments. RV-A16 (VR-283, ATCC) were propagated by infection of 80–90% confluent H1 HeLa cells. Cell lysates were collected upon the death of 90% of the monolayer.

### Virus purification

Cell lysates were collected upon death of 90% of the monolayer, determined by light microscopy, and cell debris was pelleted by centrifugation at 900 x g for 5 min at 25 °C and resuspended in 1% (v/v) NP-40 in PBS. Cell debris was then freeze-thawed three times at -20 °C and then pelleted by centrifugation at 3000 x g for 10 min at 22 °C. Supernatants were pooled and precipitated by incubation in 50% saturated ammonium sulfate precipitation at 4 °C overnight, followed by centrifugation at 4,000 x g for 1 h at 4 °C. Supernatant was discarded, and the precipitate was resuspended in PBS with 1% (v/v) NP-40. Precipitate was pelleted through a 30% (w/v) sucrose cushion at 120,000 × g (using a Beckman SW32 Ti rotor) for 3.5 h at 4°C. The pellet was resuspended in PBS and clarified by differential centrifugation. The supernatant was purified through a 15–45% (w/v) sucrose density gradient by ultracentrifugation at 160,000 × g (using a Beckman SW40 Ti rotor) for 2.5 h at 4 °C. Gradients were fractionated, and peak fractions corresponding to virions were identified by OD at 260/280 nm.

### ELISA

96-well plates (Fisher Scientific, 10511894) were coated overnight with 50 µL capture antigen (peptides 2.4mM, peptide/display vector 2.4 mM/2.4 mM, diluted in 0.05 M carbonate-bicarbonate buffer) overnight at 4 °C. Wells were washed 5 times with PBS with 0.1% (v/v) Tween (PBS-T) and blocked with 5% blocking buffer (5% (w/v) skimmed milk (Marvel) + PBS-T) for 1 h at 22 °C. All capture antigen wells were incubated with sera or controls diluted in 1% skimmed milk in PBS-T at either a 1:160 dilution or twofold serial dilutions spanning 1:80–1:640 for 1 h at 22 °C. Wells were washed with PBS-T. Mouse sera sample incubated with goat anti-mouse IgG (H + L) HRP-conjugated secondary antibodies (31430, Thermo) for 1 h at 22 °C. anti-VP4 neutralising control sera (positive control) was incubated with donkey anti-sheep IgG (H + L) HRP-conjugated secondary antibodies (A16041, Thermo) for 1 h at 22 °C. For isotyping experiments, secondary antibodies were acquired from a Mouse Antibody Isotyping Kit (ISO2–1KT, Merck). Samples were incubated with different goat anti-mouse secondary antibodies (anti-mouse IgA, anti-mouse IgM, anti-mouse IgG1, anti-mouse IgG2a, anti-mouse IgG2b, anti-mouse IgG3) for 1 h at 22 °C, followed by incubation with donkey anti-sheep IgG (H + L) HRP-conjugated secondary antibodies (A16041, Thermo) for 1 h at 22 °C. Wells were washed with PBS-T and incubated with 100 µL 1-Step Ultra TMB-ELISA (34029, Thermo) for 8 min and stopped with 0.16 M H_2_SO_4_. Colourimetric change was quantified using a GloMax microplate reader at OD_450_.

### Virus neutralisation test (VNT)

Serially diluted sera were incubated with 1.4 × 10^4^ PFU/mL RV-A16 in 4% (v/v) FBS + 1% (v/v) penicillin/streptomycin DMEM at 4°C overnight. Sera + RV-A16 mixtures were then added to 96-well plates containing 80–90% confluent H1-HeLa cells in 4% (v/v) FBS + 1% (v/v) penicillin/streptomycin in DMEM. Plates were incubated at 37 °C with 5% (v/v) CO_2_ for 3 days. Wells were then stained with 100 µL crystal violet fixing and staining solution (2.5 g crystal violet + 50 mL ethanol + 380.6 mL PBS + 70mL 36% (w/v) paraformaldehyde) for 10 min at 22 °C. Plates were washed with water and allowed to air dry at 22 °C. Wells were incubated with 100 µL 1% (w/v) sodium dodecyl sulfate (SDS) in dH_2_O for 15 min at 22 °C in an orbital shaker. Resuspended crystal violet was quantified using a GloMax microplate reader at OD_560_ as previously described in [44].

### NMR spectroscopy

Peptide samples for NMR contained 1 mM peptide solubilized in 10 mM MES buffer at pH 6.0 and 50 mM NaCl. Spectra were recorded at 22.3T and 20 °C, using a Bruker Avance III HD spectrometer with a high-sensitivity 5 mm TCI Cryoprobe. Resonance assignments for ^1^H were obtained first from 2D homonuclear NOESY and TOCSY spectra. The amide ^15^N and ^13^Cα chemical shifts were then assigned by correlations between the amide 1H and 1Hα chemical shifts in a ^1^H,^13^C HSQC and a ^1^H,^15^N HMQC, respectively, which took advantage of the natural abundance of the ^13^C and ^15^N isotopes. Secondary structure propensities were calculated using SSP with 1^H^α and ^13^Cα as the input chemical shifts [48].

### Statistical analysis

Statistical analysis and data visualisation were performed using a licensed GraphPad Prism (version 9.1.2). OD_450_ and OD_560_ obtained were subtracted from blank control wells and plotted as such. Statistical analysis was performed using one-way or two-way ANOVA, as appropriate, with the significance threshold set at α = 0.05. Post-hoc analysis was performed based on the type of comparisons performed. For data compared to a single control setup, we performed Dunnett’s test. For data where the change in signal per mouse is of interest, we performed paired t-tests. For data where multiple comparisons are performed, we utilised two-way ANOVA and post-hoc analysis was performed using Sidak’s multiple comparison test. Data were presented as mean ± 1 S.D. when applicable.
