# Supplementary material for: Induction of neutralising antibodies against conserved rhinovirus capsid protein VP4 is dependent on presentation of VP4 peptide immunogens in a virus-like conformation
Source: PLoS Pathog. 2026 Jul 16;22(7):e1014416. doi: 10.1371/journal.ppat.1014416 (PMC13405067; doi:10.1371/journal.ppat.1014416)
Supplement: Attachment — Submitted filename: Response-to-Reviews.docx [file ppat.1014416.s001.docx]

PPATHOGENS-D-25-03230
Induction of neutralising antibodies against conserved rhinovirus capsid protein VP4 is dependent on presentation of VP4 in a virus-like conformation

**Response to Reviewers**

Dear Editors and Reviewers,

Thank you for the review of this article and opportunity to respond. Please find below a detailed rebuttal against all concerns from the review.

Track changed and unmarked version of the revised manuscript have been uploaded. Line numbers given below relate to the track changes version of the revised manuscript.

Regards,

James Kelly and Toby Tuthill

**Reviewers' Comments:**

**Part II – Major Issues: Key Experiments Required for Acceptance**

**Reviewer #1**

- How was group size n=5 determined. statistical analysis?

***Response:*** The following text has been added to the materials and methods (Line 773-780):

The number of animals used was intended to be the lowest possible to generate robust data and achieve the scientific objectives of the experiment. Group sizes of 5 were based on variation in antibody titres seen in previous studies. Power calculations showed that such a group size was likely to produce statistically relevant data. For example, immunising mice with picornavirus derived peptides we would expect a 4 log2 difference in virus specific antibody titres with a standard deviation of 1.4 log2. A group size of five mice will detect differences of these magnitudes and with these standard deviations with 90% power and 95% confidence (computed using the power.t.test function in R).

- When describing the results of the VNT presented in Figure 3b the authors state that there is non-specific background neutralization in some animals. This needs to be clarified. Why and how would this happen? Is the VNT not robust enough? It would be important to understand this because they use this as Cut-off level for the VNT. It would be necessary to see if this is upon immunization. The authors should provide VNT data from these animals from the pre-immune serum and the subsequent bleedings.

***Response:*** Thank you for highlighting this, we have tried to clarify the text at line 217-225 as follows: This indicates that mock sera do not protect cells from infection with RV-A16. However, a single serum sample in SpyVLP-5 (vector only) and a single sample in the KLH (vector only) groups appeared to show a low level of non-specific neutralisation (Figure 3B). To aid visualisation of the data, we therefore set a cut off (0.7 OD) for neutralisation for any values that were above this non-specific level. We are not able explain the presence of low levels of non-specific neutralising antibodies in these individual vector only immunised animals but it was only observed in 2 out of a total of 15 vector only control animals. It would have been interesting to analyse neutralisation in the pre-immune sera, but only the final bleed samples provided sufficient material for analysis by VNT.

- Figure 3b needs to get highly improved. At this point one cannot identify what is shown in the figure also ith lack of information from the Figure legend. What is the 0.5 dose, the 5 and the KLH? Colors might be very helpful. Also black dots on black bars cannot be seen. It seems like this figure was made by another person because the other figures are in color. Taken together, without a highly improved figure the paragraph described in the results cannot be reviewed properly.

**Response:** Apologies that part of the figure was not displayed as intended and the X axis labels were omitted. This has been corrected. We have also changed all data points to be open circles to make clearer. We hope with these changes this figure is now clear*.*

**Reviewer #2:**

- KLH conjugate DLS measurements would be good to show.

**Response:** We appreciate this request but unfortunately do not have this data. Our rationale at the time was that given the tendency of the myristolyated peptides to cause aggregation (as seen in the work with the SpyCatcher conjugation) and the general tendency for KLH conjugates to often aggregate without negative impact on generation of responses, we assumed aggregation was likely and did not investigate the KLH conjugates by DLS. The KLH conjugates were not intended to be a direct comparison with the SpyCatcher immunogens, only as a general control for VP4 responses (we and others have successfully used VP4 peptide-KLH previously and we have added references at line 185 to make this point), so we thought the value of DLS analysis would be limited. We could re-make the KLH conjugates and analyse them but without repeating the immunisations with the analysed material we also feel this would have limited value.

**Reviewer #3:**

I have several comments on the methods and the results obtained:

1.) If I understood correctly, the conjugation of VP4 peptides to a SpyVLPs was verified by SDS-PAGE as shown in Figure 2. But has the conjugation for the KLH been checked? There are several methods, such as the Ellman reagent test, that could be used. Is it possible that the number of peptides conjugated to different display systems is different? Can that be compared and/or standardized?

- has the conjugation for the KLH been checked?

**Response:** We appreciate this request but unfortunately do not have data for KLH conjugation efficiency. We checked the conjugation of peptides to Spy VLPs because this is a relatively novel and experimental system for generating immunogens. We did not check the conjugation of peptides to KLH because this is a well established approach for generating immunogens. The KLH conjugates were not intended to be a direct comparison with the SPyCatcher immunogens, only as a general control for VP4 responses (we and others have successfully used VP4 peptide-KLH previously and we have added references at line 185 to make this point), so we thought the value of knowing the conjugation efficiency would be limited. We could re-make the KLH conjugates and analyse them but without repeating the immunisations with the analysed material we also feel this would have limited value.

- Is it possible that the number of peptides conjugated to different display systems is different? Can that be compared and/or standardized?

**Response:** Conjugation within the SpyCatcher group was broadly similar. The level of conjugation between the SpyCatcher and the KLH may be different but the KLH conjugates were general controls and not intended to be a qualitative comparison to SpyCatcher. Therefore as described above (in response to reviewer 2), we thought a detailed analysis of conjugation for the KLH was of limited value.

2.) The authors used groups of 5 mice to be immunized. Considering so many groups included in the study was the number of 5 mice sufficient to get a power of at least 80%?

**Response:** Our response to this question is the same as to reviewer 1:

The following text has been added to the materials and methods (Line 773-780):

The number of animals used was intended to be the lowest possible to generate robust data and achieve the scientific objectives of the experiment. Group sizes of 5 were based on variation in antibody titres seen in previous studies. Power calculations showed that such a group size was likely to produce statistically relevant data. For example, immunising mice with picornavirus derived peptides we would expect a 4 log2 difference in virus specific antibody titres with a standard deviation of 1.4 log2. A group size of five mice will detect differences of these magnitudes and with these standard deviations with 90% power and 95% confidence (computed using the power.t.test function in R).

**Response:** Why did the authors use only female Balb/c mice?

At the time of study design (2021), this was the preferred approach to improve animal welfare by minimising potential aggression within a group containing multiple males. The following statement has been added at line 743: “Only female animals were used in order to minimise potential aggression within groups”. Since then, study design has changed to include both male and female animals.

3.) Figure 2: the development of the VP4-specific responses in mice was tested only to the longest VP4 peptide alone. Why the authors did not investigate the antibody responses to the corresponding immunogen used to immunize the mice (SpyVLP and KLH conjugated peptides)? This would have been more informative if it had been taken into account that the first 1-15 amino acids of VP4 can be detected only after conjugation using a display system.

**Response:** Thanks for this comment. We assume this comment is related to Figure 4 (not Figure 2). We have altered the text at lines 248 – 256 to hopefully clarify the rationale for using the peptide capture antigen:

“Now that we had established that certain end-point sera were neutralising, we wanted to determine how the overall strength of peptide-specific immune response developed during the immunisation experiment. For the initial investigation we analysed sera from each bleed time point by ELISA using 1-45-VP4 peptide as the capture antigen (coated directly on to the plate). The complete immunogen was not used as a capture antigen to avoid detection of responses directed against the carrier. As all immunogens in this study contain a peptide sequence within the 1-45-VP4 peptide, we therefore expected the 1-45-VP4 peptide to capture all VP4-specific antibodies produced in this study.”

4.) Figure 3: The VNT was used to investigate the ability of induced VP4-specific antibodies to neutralize RV-A16 infection in HeLa cells. However, it is unclear to me what the negative controls were—PBS or serum from mice immunized with PBS? What OD values were measured for the wells containing medium alone? Since the neutralization titers were not very high, the results could be confirmed by including pre-immunization serum (if available) or normal mouse serum, which sometimes also gives positive results at a dilution of 1:2. Please also add the names of the mouse groups to the x-axis in the graph and change the colours to make the points more visible.

**Response:** Pre-immune sera was not available in sufficient quantity to carry out any meaningful analysis. This has been added to the manuscript at line 223-225. We believe we have controlled for the neutralisation in serum dilutions of 1:2 by including the vector only sera at 1:2 dilution to give an estimated cut-off for what we consider to be neutralising. Apologies that part of the figure was not displayed and the X axis labels were omitted. Negative controls for the immunisation were the vector only sera. PBS is the ‘medium alone’ control for the ELISA and is included in the figure. We have also changed all data points to be open circles to make clearer. We hope with the inclusion of the labels this figure is now clear.

**Part III – Minor Issues: Editorial and Data Presentation Modifications**
Please use this section for editorial suggestions as well as relatively minor modifications of existing data that would enhance clarity.

**Reviewer #1:**

- The authors identify the importance of the conformation of the peptide. Is it possible to model the 1-15 VP4 peptide in the capsid integrated form?

**Response:** We can see VP4 as an internal capsid component in several picornavirus structures derived by X-ray crystallography or cryo-EM. However in the context of the current manuscript, the relevant conformation for the N-terminus of VP4 is at the surface of the capsid. We would love to see a virus structure showing VP4 in this externalised conformation but no structure of this form has been determined yet, likely due to the transient nature of VP4 exposure at the capsid surface. We are therefore also unable to model the N-terminus of VP4 in this conformation because the site of egress to the outside of the capsid is not known.

- For Table 1 would be good to indicate the first 15aa in bold and/or tag in italic that is easy to see similarities and differences.

**Response:** Thanks for this suggestion, the table has been modified.

**Reviewer #2:**

- Lines 171-178: It is stated that KLH aggregation is not thought to prevent a functional response. Please, give a reference for this.

**Response:** References have been added at line 186, to support the concept that aggregation of KLH does not affect induction of antibody responses.

- However, DLS of the KLH constructs would be good to show.

**Response:** DLS of the KLH conjugates was not carried out with the rationale already given in the paper (aggregation is not thought to be a problem for presentation by KLH). The exact conjugates used for the current study no longer exist; we could remake more of these conjugates and characterise by DLS, but unless this was done for the same batch of material used for the immunisation, we feel this would have limited value.

- As the myristoylated constructs were not used in the major experiments, even for KLH, the line 176-7 could be omitted as futile.

**Response:** We are unsure about this comment. Myristoylated VP4 peptides were used for conjugation to KLH as described in the existing text (from line 181-182 in revised manuscript) “…myristoylated VP4 peptides were also displayed using the well-established KLH peptide presentation system…)

Some spelling errors are present in the MS. Please, correct those.

**Response:** We have attempted to find and correct all spelling errors and these are shown in the track changes manuscript.

722 Define what plates were used. **Response:** Thanks, done.

Figure 3: In the figure it is not indicated what results associate with SpyVLP in towodoses or KLH. Add those.

**Response:** This figure has been modified.

**Reviewer #3:**

I have also some minor comments:
1.) Title – I found the title a little bit misleading since the authors did not use the complete RV capsid proteins but 3 N terminal peptides of different length which might have influenced the conformation upon conjugation to a display system. Please rephrase to be more precise.

Thank you for this suggestion.

**Response:** Revised title: Induction of neutralising antibodies against conserved rhinovirus capsid protein VP4 is dependent on presentation of VP4 peptide immunogens in a virus-like conformation

2.) Introduction – I would prefer the term "RV strains" to be used in the manuscript instead of "serotypes" as the new types are no longer identified by serology but by genetic homology.

**Response:** We discussed amongst the authors the use of ‘serotype’. We agree that serotype is not applicable to newer viruses identified by genetic homology but feel that in the context of emphasising the potential for cross-reactive responses it is still a useful definition. As a compromise we have altered the description of serotypes in the abstract to distinguish between strains and serotypes. (Line 21-23)

Furthermore, the species RVA, RVB, and RVC should be designated as RV-A, RV-B, and RV-C.

**Response:** Thanks, this has been changed.

Line 103: hepatitis B virus should be written.

**Response:** Thanks, this has been changed.

3.) Results:
Line 217 – Table 1 contains the amino acid sequences of the peptides and there is no information regarding their neutralising capacity. I would rather delete it from the text.

**Response:** Thank you, this has been deleted.

Line 343: why 4 sera and not 5 have been tested here?

**Response:** All five sera were tested but one had reactivity that mapped to the lysines (included to enhance solubility) and therefore only the remaining four sera were described in detail.

Line 428: ….to contain **Response:** done
Line : 442: again Table 1 lists only the amino acid sequences of the peptides used in the study. **Response:** done
Line 866: please delete ****p<0.00005 (not shown on the figure). **Response:** Done

4.) Methods:
Line 723: In the methods section describing the ELISA tests, it is mentioned that ICAM-1 was applied to the ELISA plate and incubated with the RV-16 virus. Where can I see the results of these experiments? How was the virus detected?

**Response:** Thanks for finding this. This was an approach that was not used in this manuscript and was included in error. This has been removed from the revised manuscript.

5.) Discussion - the authors should speculate more about when the induced antibodies can neutralize the virus during a natural infection. Since VP4-specific antibodies can neutralize the virus, as mentioned in the introduction, by preventing the interaction between VP4 and the endosomal membrane, the question arises as to how these antibodies enter the cells. Please provide your opinion.

**Response:** Thank you, this is a good idea. We have added this paragraph to the discussion at line 588 - 592: “Understanding the mechanism of neutralisation by VP4-specific antibodies would also be very interesting. Antibodies may bind to VP4 N-terminal epitopes during transient exposure of the VP4 N-terminus during capsid breathing. This could neutralise virus by hindering receptor binding or the immune complex may still be able to bind to receptor and be internalised but with reduced ability to interact with and permeabilise the endocytosed vesicle.”
