# Supplementary material for: Induction of neutralising antibodies against conserved rhinovirus capsid protein VP4 is dependent on presentation of VP4 peptide immunogens in a virus-like conformation
Source: PLoS Pathog. 2026 Jul 16;22(7):e1014416. doi: 10.1371/journal.ppat.1014416 (PMC13405067; doi:10.1371/journal.ppat.1014416)
Supplement: Peer review history

# Peer review: Decision Letter 0

**DOI:** 10.1371/journal.ppat.1014416.r001

**Authors:** Caroline Tapparel, Ashley L. St. John

4 Mar 2026

-->PPATHOGENS-D-25-03230

Induction of neutralising antibodies against conserved rhinovirus capsid protein VP4 is dependent on presentation of VP4 in a virus-like conformation

PLOS Pathogens

Dear Dr. Kelly,

Thank you for submitting your manuscript to PLOS Pathogens. After careful consideration, we feel that it has merit but does not fully meet PLOS Pathogens's publication criteria as it currently stands. Therefore, we invite you to submit a revised version of the manuscript that addresses the points raised during the review process.

Please submit your revised manuscript by April 3rd. If you will need more time than this to complete your revisions, please reply to this message or contact the journal office at plospathogens@plos.org. When you're ready to submit your revision, log on to https://www.editorialmanager.com/ppathogens/ and select the 'Submissions Needing Revision' folder to locate your manuscript file.

Please include the following items when submitting your revised manuscript:

* A letter that responds to each point raised by the editor and reviewer(s). You should upload this letter as a separate file labeled 'Response to Reviewers'. This file does not need to include responses to any formatting updates and technical items listed in the 'Journal Requirements' section below.

* A marked-up copy of your manuscript that highlights changes made to the original version. You should upload this as a separate file labeled 'Revised Manuscript with Track Changes'.

* An unmarked version of your revised paper without tracked changes. You should upload this as a separate file labeled 'Manuscript'.

If you would like to make changes to your financial disclosure, competing interests statement, or data availability statement, please make these updates within the submission form at the time of resubmission. Guidelines for resubmitting your figure files are available below the reviewer comments at the end of this letter.

We look forward to receiving your revised manuscript.

Kind regards,

Caroline Tapparel

Academic Editor

PLOS Pathogens

Ashley St. John

Section Editor

PLOS Pathogens

Sumita Bhaduri-McIntosh

Editor-in-Chief

PLOS Pathogens

orcid.org/0000-0003-2946-9497

Michael Malim

Editor-in-Chief

PLOS Pathogens

orcid.org/0000-0002-7699-2064

**Journal Requirements:**

1) Please ensure that the CRediT author contributions listed for every co-author are completed accurately and in full.

At this stage, the following Authors/Authors require contributions: James T Kelly, Giann K Dellosa, Joseph K Newman, Adel A. M. Mohamed, Julia Aniscenko, Sebastian L Johnston, Jason R Schnell, and Toby J Tuthill. Please ensure that the full contributions of each author are acknowledged in the "Add/Edit/Remove Authors" section of our submission form.

The list of CRediT author contributions may be found here: https://journals.plos.org/plospathogens/s/authorship#loc-author-contributions

2) We ask that a manuscript source file is provided at Revision. Please upload your manuscript file as a .doc, .docx, .rtf or .tex. If you are providing a .tex file, please upload it under the item type u2018LaTeX Source Fileu2019 and leave your .pdf version as the item type u2018Manuscriptu2019.

3) We do not publish any copyright or trademark symbols that usually accompany proprietary names, eg ©,  ®, or TM  (e.g. next to drug or reagent names). Therefore please remove all instances of trademark/copyright symbols throughout the text, including:

- TM on page: 12.

4) Please upload all main figures as separate Figure files in .tif or .eps format. For more information about how to convert and format your figure files please see our guidelines:

https://journals.plos.org/plospathogens/s/figures

5) Some material included in your submission may be copyrighted. According to PLOSu2019s copyright policy, authors who use figures or other material (e.g., graphics, clipart, maps) from another author or copyright holder must demonstrate or obtain permission to publish this material under the Creative Commons Attribution 4.0 International (CC BY 4.0) License used by PLOS journals. Please closely review the details of PLOSu2019s copyright requirements here: PLOS Licenses and Copyright. If you need to request permissions from a copyright holder, you may use PLOS's Copyright Content Permission form.

Please respond directly to this email and provide any known details concerning your material's license terms and permissions required for reuse, even if you have not yet obtained copyright permissions or are unsure of your material's copyright compatibility. Once you have responded and addressed all other outstanding technical requirements, you may resubmit your manuscript within Editorial Manager.

Potential Copyright Issues:

i) Figure 3a. Please confirm whether you drew the images / clip-art within the figure panels by hand. If you did not draw the images, please provide (a) a link to the source of the images or icons and their license / terms of use; or (b) written permission from the copyright holder to publish the images or icons under our CC BY 4.0 license. Alternatively, you may replace the images with open source alternatives. See these open source resources you may use to replace images / clip-art:

- https://commons.wikimedia.org

- https://openclipart.org/.

6) We note that your Data Availability Statement is currently as follows: "All data are in the manuscript". Please confirm at this time whether or not your submission contains all raw data required to replicate the results of your study. Authors must share the “minimal data set” for their submission. PLOS defines the minimal data set to consist of the data required to replicate all study findings reported in the article, as well as related metadata and methods (https://journals.plos.org/plosone/s/data-availability#loc-minimal-data-set-definition).

For example, authors should submit the following data:

1) The values behind the means, standard deviations and other measures reported;

2) The values used to build graphs;

3) The points extracted from images for analysis..

Authors do not need to submit their entire data set if only a portion of the data was used in the reported study.

If your submission does not contain these data, please either upload them as Supporting Information files or deposit them to a stable, public repository and provide us with the relevant URLs, DOIs, or accession numbers. For a list of recommended repositories, please see https://journals.plos.org/plosone/s/recommended-repositories.

If there are ethical or legal restrictions on sharing a de-identified data set, please explain them in detail (e.g., data contain potentially sensitive information, data are owned by a third-party organization, etc.) and who has imposed them (e.g., an ethics committee). Please also provide contact information for a data access committee, ethics committee, or other institutional body to which data requests may be sent. If data are owned by a third party, please indicate how others may request data access.

7) Please amend your detailed Financial Disclosure statement. This is published with the article. It must therefore be completed in full sentences and contain the exact wording you wish to be published.

1) State the initials, alongside each funding source, of each author to receive each grant. For example: "This work was supported by the National Institutes of Health (####### to AM; ###### to CJ) and the National Science Foundation (###### to AM)."

2) State what role the funders took in the study. If the funders had no role in your study, please state: "The funders had no role in study design, data collection and analysis, decision to publish, or preparation of the manuscript.".

Note: If the reviewer comments include a recommendation to cite specific previously published works, please review and evaluate these publications to determine whether they are relevant and should be cited. There is no requirement to cite these works unless the editor has indicated otherwise.

**Reviewers' Comments:**

Reviewer's Responses to Questions

**Part I - Summary**

Please use this section to discuss strengths/weaknesses of study, novelty/significance, general execution and scholarship.

Reviewer #1: Kelly and colleagues investigate RV VP4 derived peptides for their potential to induce neutralizing antibodies upon immunization. Using previous studies as directive they focused on the N-terminal part of the VP4. The authors provide detailed information and characterization on the used VLP coupled-VP4 peptides, they show VNT data after immunizing mice and report detailed analysis of the obtained serum samples in terms of identifying the neutralizing epitope. The data provided might indicate that the conformation of the VP4 peptide is important for the neutralization.

There are certain point that need to be addressed, which would improve the manuscript to make it suitable for publication in PLOS Pathogens.

Reviewer #2: This is a thorough study concerning the antigenicity of VP4 and will help with future planning for future vaccine or antiviral strategies.

Reviewer #3: The article by Kelly et al. describes efforts to elucidate the immunogenicity of three RV N-terminal peptides of different sizes and presentation systems. The topic is very important because there is currently no vaccine or antiviral agent against RV infections. The question of what can influence the neutralization capacity of the induced antibodies is also of fundamental importance.

The authors first produced the N-terminal VP4 peptides with three different lengths, which were presented on either SpyCatcher VLPs or KLH, then immunized the mice, and analyzed the ability of the induced VP4-specific antibodies to neutralize RV infections of the cultured HeLa cells. They found that only antibodies directed against the shortest peptide (1-15aa) could protect HeLa cells from RV infection. Surprisingly, these antisera did not react with any of the free peptides, but only when conjugated with a display system. This was a completely unexpected observation, which was then explained by the different conformation of the antigens.

**********

**Part II – Major Issues: Key Experiments Required for Acceptance**

Please use this section to detail the key new experiments or modifications of existing experiments that should be absolutely required to validate study conclusions.

Generally, there should be no more than 3 such required experiments or major modifications for a "Major Revision" recommendation. If more than 3 experiments are necessary to validate the study conclusions, then you are encouraged to recommend "Reject".

Reviewer #1: *) For the immunizations the authors used n=5 per group. How was this number determined? Which and how statistical analysis was used to identify the group size to get significant results. All information should be included in the methods section.

*) When describing the results of the VNT presented in Figure 3b the authors state that there is non-specific background neutralization in some animals. This needs to be clarified. Why and how would this happen? Is the VNT not robust enough? It would be important to understand this because they use this as Cut-off level for the VNT. It would be necessary to see if this is upon immunization. The authors should provide VNT data from these animals from the pre-immune serum and the subsequent bleedings.

*) Figure 3b needs to get highly improved. At this point one cannot identify what is shown in the figure also ith lack of information from the Figure legend. What is the 0.5 dose, the 5 and the KLH? Colors might be very helpful. Also black dots on black bars cannot be seen. It seems like this figure was made by another person because the other figures are in color. Taken together, without a highly improved figure the paragraph described in the results cannot be reviewed properly.

Reviewer #2: KLH conjugate DLS measurements would be good to show.

Reviewer #3: I have several comments on the methods and the results obtained:

1.) If I understood correctly, the conjugation of VP4 peptides to a SpyVLPs was verified by SDS-PAGE as shown in Figure 2. But has the conjugation for the KLH been checked? There are several methods, such as the Ellman reagent test, that could be used. Is it possible that the number of peptides conjugated to different display systems is different? Can that be compared and/or standardized?

2.) The authors used groups of 5 mice to be immunized. Considering so many groups included in the study was the number of 5 mice sufficient to get a power of at least 80%? Why did the authors use only female Balb/c mice?

3.) Figure 2: the development of the VP4-specific responses in mice was tested only to the longest VP4 peptide alone. Why the authors did not investigate the antibody responses to the corresponding immunogen used to immunize the mice (SpyVLP and KLH conjugated peptides)? This would have been more informative if it had been taken into account that the first 1-15 amino acids of VP4 can be detected only after conjugation using a display system.

4.) Figure 3: The VNT was used to investigate the ability of induced VP4-specific antibodies to neutralize RV-A16 infection in HeLa cells. However, it is unclear to me what the negative controls were—PBS or serum from mice immunized with PBS? What OD values were measured for the wells containing medium alone? Since the neutralization titers were not very high, the results could be confirmed by including pre-immunization serum (if available) or normal mouse serum, which sometimes also gives positive results at a dilution of 1:2. Please also add the names of the mouse groups to the x-axis in the graph and change the colours to make the points more visible.

**********

**Part III – Minor Issues: Editorial and Data Presentation Modifications**

Please use this section for editorial suggestions as well as relatively minor modifications of existing data that would enhance clarity.

Reviewer #1: *) The authors identify the importance of the conformation of the peptide. Is it possible to model the 1-15 VP4 peptide in the capsid integrated form?

*) For Table 1 would be good to indicate the first 15aa in bold and/or tag in italic that is easy to see similarities and differences.

Reviewer #2: Lines 171-178: It is stated that KLH aggregation is not thought to prevent a functional response. Please, give a reference for this. However, DLS of the KLH constructs would be good to show. As the myristoylated constructs were not used in the major experiments, even for KLH, the line 176-7 could be omitted as futile.

Some spelling errors are present in the MS. Please, correct those.

722 Define what plates were used.

Figure 3: In the figure it is not indicated what results associate with SpyVLP in towodoses or KLH. Add those.

Reviewer #3: I have also some minor comments:

1.) Title – I found the title a little bit misleading since the authors did not use the complete RV capsid proteins but 3 N terminal peptides of different length which might have influenced the conformation upon conjugation to a display system. Please rephrase to be more precise.

2.) Introduction – I would prefer the term "RV strains" to be used in the manuscript instead of "serotypes" as the new types are no longer identified by serology but by genetic homology. Furthermore, the species RVA, RVB, and RVC should be designated as RV-A, RV-B, and RV-C.

Line 103: hepatitis B virus should be written.

3.) Results:

Line 217 – Table 1 contains the amino acid sequences of the peptides and there is no information regarding their neutralising capacity. I would rather delete it from the text.

Line 343: why 4 sera and not 5 have been tested here?

Line 428: ….to contain

Line : 442: again Table 1 lists only the amino acid sequences of the peptides used in the study.

Line 866: please delete ****p<0.00005 (not shown on the figure).

4.) Methods:

Line 723: In the methods section describing the ELISA tests, it is mentioned that ICAM-1 was applied to the ELISA plate and incubated with the RV-16 virus. Where can I see the results of these experiments? How was the virus detected?

5.) Discussion - the authors should speculate more about when the induced antibodies can neutralize the virus during a natural infection. Since VP4-specific antibodies can neutralize the virus, as mentioned in the introduction, by preventing the interaction between VP4 and the endosomal membrane, the question arises as to how these antibodies enter the cells. Please provide your opinion.

**********

PLOS authors have the option to publish the peer review history of their article (what does this mean?). If published, this will include your full peer review and any attached files.

If you choose “no”, your identity will remain anonymous but your review may still be made public.

**Do you want your identity to be public for this peer review?**  For information about this choice, including consent withdrawal, please see our Privacy Policy.

Reviewer #1: No

Reviewer #2: No

Reviewer #3: No

[NOTE: If reviewer comments were submitted as an attachment file, they will be attached to this email and accessible via the submission site. Please log into your account, locate the manuscript record, and check for the action link "View Attachments". If this link does not appear, there are no attachment files.]

**Figure resubmission:**

-->While revising your submission, we strongly recommend that you use PLOS’s NAAS tool (https://ngplosjournals.pagemajik.ai/artanalysis) to test your figure files. NAAS can convert your figure files to the TIFF file type and meet basic requirements (such as print size, resolution), or provide you with a report on issues that do not meet our requirements and that NAAS cannot fix.

After uploading your figures to PLOS’s NAAS tool - https://ngplosjournals.pagemajik.ai/artanalysis, NAAS will process the files provided and display the results in the "Uploaded Files" section of the page as the processing is complete. If the uploaded figures meet our requirements (or NAAS is able to fix the files to meet our requirements), the figure will be marked as "fixed" above. If NAAS is unable to fix the files, a red "failed" label will appear above. When NAAS has confirmed that the figure files meet our requirements, please download the file via the download option, and include these NAAS processed figure files when submitting your revised manuscript.

**Reproducibility:**

To enhance the reproducibility of your results, we recommend that authors of applicable studies deposit laboratory protocols in protocols.io, where a protocol can be assigned its own identifier (DOI) such that it can be cited independently in the future. Additionally, PLOS ONE offers an option to publish peer-reviewed clinical study protocols. Read more information on sharing protocols at https://plos.org/protocols?utm_medium=editorial-email&utm_source=authorletters&utm_campaign=protocols

# Author comment: Author response to Decision Letter 1

**DOI:** 10.1371/journal.ppat.1014416.r002

13 May 2026

Attachment Submitted filename: Response-to-Reviews.docx

# Peer review: Decision Letter 1

**DOI:** 10.1371/journal.ppat.1014416.r003

**Authors:** Ashley L. St. John

24 Jun 2026

Dear Dr Kelly,

We are pleased to inform you that your manuscript 'Induction of neutralising antibodies against conserved rhinovirus capsid protein VP4 is dependent on presentation of

VP4 peptide immunogens in a virus-like conformation' has been provisionally accepted for publication in PLOS Pathogens.

Before your manuscript can be formally accepted you will need to complete some formatting changes, which you will receive in a follow up email. A member of our team will be in touch with a set of requests.

Please note that your manuscript will not be scheduled for publication until you have made the required changes, so a swift response is appreciated.

IMPORTANT: The editorial review process is now complete. PLOS will only permit corrections to spelling, formatting or significant scientific errors from this point onwards. Requests for major changes, or any which affect the scientific understanding of your work, will cause delays to the publication date of your manuscript.

Should you, your institution's press office or the journal office choose to press release your paper, you will automatically be opted out of early publication. We ask that you notify us now if you or your institution is planning to press release the article. All press must be co-ordinated with PLOS.

Thank you again for supporting Open Access publishing; we are looking forward to publishing your work in PLOS Pathogens.

Best regards,

Ashley L. St. John

Section Editor

PLOS Pathogens

Caroline Tapparel

Academic Editor

PLOS Pathogens

Sumita Bhaduri-McIntosh

Editor-in-Chief

PLOS Pathogens

orcid.org/0000-0003-2946-9497

Michael Malim

Editor-in-Chief

PLOS Pathogens

orcid.org/0000-0002-7699-2064

***********************************************************

Reviewer Comments (if any, and for reference):

Reviewer's Responses to Questions

**Part I - Summary**

Please use this section to discuss strengths/weaknesses of study, novelty/significance, general execution and scholarship.

Reviewer #1: (No Response)

Reviewer #2: (No Response)

Reviewer #3: (No Response)

**********

**Part II – Major Issues: Key Experiments Required for Acceptance**

Please use this section to detail the key new experiments or modifications of existing experiments that should be absolutely required to validate study conclusions.

Generally, there should be no more than 3 such required experiments or major modifications for a "Major Revision" recommendation. If more than 3 experiments are necessary to validate the study conclusions, then you are encouraged to recommend "Reject".

Reviewer #1: (No Response)

Reviewer #2: (No Response)

Reviewer #3: (No Response)

**********

**Part III – Minor Issues: Editorial and Data Presentation Modifications**

Please use this section for editorial suggestions as well as relatively minor modifications of existing data that would enhance clarity.

Reviewer #1: (No Response)

Reviewer #2: (No Response)

Reviewer #3: (No Response)

**********

PLOS authors have the option to publish the peer review history of their article (what does this mean?). If published, this will include your full peer review and any attached files.

If you choose “no”, your identity will remain anonymous but your review may still be made public.

**Do you want your identity to be public for this peer review?**  For information about this choice, including consent withdrawal, please see our Privacy Policy.

Reviewer #1: No

Reviewer #2: No

Reviewer #3: No

# Editor report: Acceptance letter

**DOI:** 10.1371/journal.ppat.1014416.r004

**Authors:** Ashley L. St. John

Dear Dr Kelly,

We are delighted to inform you that your manuscript, "Induction of neutralising antibodies against conserved rhinovirus capsid protein VP4 is dependent on presentation of

VP4 peptide immunogens in a virus-like conformation," has been formally accepted for publication in PLOS Pathogens.

We have now passed your article onto the PLOS Production Department who will complete the rest of the pre-publication process. All authors will receive a confirmation email upon publication.

The corresponding author will soon be receiving a typeset proof for review, to ensure errors have not been introduced during production. Please review the PDF proof of your manuscript carefully, as this is the last chance to correct any scientific or type-setting errors. Please note that major changes, or those which affect the scientific understanding of the work, will likely cause delays to the publication date of your manuscript. Note: Proofs for Front Matter articles (Pearls, Reviews, Opinions, etc...) are generated on a different schedule and may not be made available as quickly.

Soon after your final files are uploaded, the early version of your manuscript, if you opted to have an early version of your article, will be published online. The date of the early version will be your article's publication date. The final article will be published to the same URL, and all versions of the paper will be accessible to readers.

For Research Articles, you will receive an invoice from PLOS for your publication fee after your manuscript has reached the completed accept phase. If you receive an email requesting payment before acceptance or for any other service, this may be a phishing scheme. Learn how to identify phishing emails and protect your accounts at https://explore.plos.org/phishing.

Thank you again for supporting open-access publishing; we are looking forward to publishing your work in PLOS Pathogens.

Best regards,

Sumita Bhaduri-McIntosh

Editor-in-Chief

PLOS Pathogens

orcid.org/0000-0003-2946-9497

Michael Malim

Editor-in-Chief

PLOS Pathogens

orcid.org/0000-0002-7699-2064
